# Supplementary material for: Patterns and drivers of Holocene moisture variability in mid-latitude eastern North America
Source: Nat Commun. 2025 Apr 15;16:3582. doi: 10.1038/s41467-025-58685-7 (PMC12000431; doi:10.1038/s41467-025-58685-7)
Supplement: Supplementary file 1 — Supplementary Information [file 41467_2025_58685_MOESM1_ESM.pdf]

Supplementary information

Patterns and drivers of Holocene moisture variability in mid-latitude eastern North America

J. Sakari Salonen, Frederik Schenk, John W. Williams, Bryan Shuman, Ana L. Lindroth Dauner, Sebastian Wagner, Johann Jungclaus, Qiong Zhang, Miska Luoto

Supplementary Table 1. Fossil data sites used. Indicated for each site are the site cluster (MW = Midwest, GL = Great Lakes, NE = Northeast), site name, the dataset ID in the Neotoma database[1] (https://www.neotomadb.org), modern annual water balance (mm) and July mean temperature (T_{jul}, in °C) values[2], the number of pollen samples and ^{14}C datings (with sites selected for the high-resolution reconstructions marked with asterisks), the span of the age-depth model (in calibrated ka), the name of the age-depth model used in Neotoma, and references to the original publications.

Cluster	Name, State/Province	Neotoma ID	Lat.	Lon.	Water balance	T_{jul}	High res.	Sample #	Date #	Age range	Age model	References
MW	West Okoboji Lake, IA	1824	43.333	−95.2	183	22.4		110	10	0–15.2	Wang et al. 2019	[3]
MW	Clear Lake, IA	489	43.125	−93.43	237	22.5		53	9	0–12.2	Neotoma 1	[4]
MW	Myrtle Lake, MN	1786	47.983	−93.39	242	19.4		82	5	0–12.7	PalEON-STEPPS	[5-7]
MW	Little Bass Lake, MN	1639	47.286	−93.6	230	20.3		37	5	0–12.3	PalEON-STEPPS	P.C. Swain, unpub.
MW	Irvin Lake, MN	1153	47.136	−93.64	226	20.4		51	7	0–15.9	PalEON-STEPPS	[8]
MW	Reidel Lake, MN	2053	46.212	−95.28	152	21.7		51	6	0–13.7	PalEON-STEPPS	[9,10]
MW	Billy’s Lake, MN	275	46.271	−94.55	199	21.4		77	6	0–12.6	PalEON-STEPPS	[11-15]
MW	Deep Lake, MN	15403	47.684	−95.4	169	19.6		62	6	0.2–10.2	PalEON-STEPPS	[16,17]
MW	Wolsfeld Lake, MN	3031	45.005	−93.57	191	22.8		68	10	0–13.8	Neotoma 1	[18,19]
MW	Lily Lake, MN	1633	45.048	−92.82	206	21.9		31	7	0–14.8	PalEON-STEPPS	[20]
MW	Rutz Lake, MN	2314	44.871	−93.86	189	22.9		39	7	0–13.6	PalEON-STEPPS	[21,22]
MW	Sharkey Lake, MN	13029	44.592	−93.41	217	22.6	*	165	11	0.1–12.0	PalEON-STEPPS	[23]
MW	Kimble Pond, MN	13032	44.212	−93.84	186	22.9	*	236	9	0–12.3	Neotoma 1	[23]
MW	Steel Lake, MN	17396	46.973	−94.68	215	20.5	*	125	26	0–12.1	Neotoma 2	[24-27]
MW	Kettle Lake, ND	20194	48.607	−103.6	−106	20.7	*	551	53	0–12.9	Neotoma 1	[28-30]
MW	Moon Lake, ND	1768	46.856	−98.16	−8	21.4	*	170	14	0–14.0	Neotoma 1	[31]
MW	Devils Lake, ND	15136	48.086	−98.93	−18	20.5		64	5	0–14.3	Neotoma 1	[32]
MW	Jones Lake, Manitoba	15138	49.45	−99.29	88	19.3	*	127	13	0–11.2	Neotoma 1	[33]
MW	E Lake, Manitoba	3125	50.691	−99.66	158	17.7		57	8	0–13.5	NAPD 1	[34-36]
MW	Lake 239, Ontario	26606	49.663	−93.72	267	19.1		47	7	0–10.2	Neotoma 1	[37]
GL	Nelson Lake, IL	48685	41.836	−88.38	318	22.7		76	16	0–18.1	Grimm 2020	[38-42]
GL	Spicer Lake, IN	19937	41.757	−86.52	378	22.8		145	15	0–15.3	Wang et al. 2016	[43]
GL	Camp 11 Lake, MI	332	46.667	−88.02	434	18.1		37	11	0–11.6	PalEON-STEPPS	[44]
GL	Canyon Lake, MI	15682	46.833	−87.92	419	18.5		68	8	0–11.8	PalEON-STEPPS	[45]
GL	Cub Lake, MI	532	44.7	−84.96	302	19.3		62	5	0–9.8	PalEON-STEPPS	[46]
GL	Demont Lake, MI	679	43.48	−85	295	21.2		74	5	0–14.2	PalEON-STEPPS	R.O. Kapp, unpub.
GL	Glimmerglass Lake, MI	13097	46.215	−89.32	417	18.4		35	5	0–11.2	PalEON-STEPPS	[47,48]
GL	Jay Lake, MI	15922	46.234	−89.28	417	18.3		33	6	0–10.2	PalEON-STEPPS	[49,50]
GL	Mud Lake, MI	15032	47.129	−88.32	405	19.1		57	7	0–9.8	PalEON-STEPPS	[51]
GL	Spirit Lake, MI	2586	46.47	−86.96	394	18.6		55	6	0–13.6	PalEON-STEPPS	[52]
GL	Wintergreen Lake, MI	2961	42.4	−85.38	308	22.4		69	8	0–15.2	PalEON-STEPPS	[53]
GL	Wolverine Lake, MI	3032	46.429	−85.66	421	18.0		42	5	0–10.5	PalEON-STEPPS	R. Futyma, unpub.
GL	Yellow Dog Pond, MI	3047	46.755	−87.94	437	18.2		37	9	0–10.3	PalEON-STEPPS	[44]
GL	Devils Lake, WI	684	43.418	−89.73	297	21.4		122	10	0–14.3	PalEON-STEPPS	[54]
GL	Emrick Lake, WI	15302	43.8	−89.59	275	21.3		92	6	0–13.1	PalEON-STEPPS	[55]
GL	Hells Kitchen Lake, WI	48818	46.186	−89.7	423	18.5		48	10	0–10.5	Neotoma 1	[56]
GL	Lake O' Pines, WI	15925	46.14	−89.26	409	18.4		35	6	0–12.3	PalEON-STEPPS	[49,50]
GL	Radtke Lake, WI	2011	43.4	−88.1	223	22.0		50	7	0–13.2	PalEON-STEPPS	[57]
NE	Mohawk Pond, CT	3493	41.811	−73.29	693	20.4		50	13	0.2–18.7	Neotoma 1	[15,58-60]
NE	West Side Pond, CT	41027	41.855	−73.26	714	20.1		48	11	0–13.4	Author submitted	[61]
NE	Mansell Pond, ME	1698	45.042	−68.73	555	19.7		110	9	0–10.2	Wang et al. 2019	[62]
NE	Mathews Pond, ME	39474	46.318	−69.06	604	18.1		76	6	0.1–15.0	Neotoma 1	[63]
NE	Poland Spring Pond, ME	1961	44.033	−70.35	643	20.7		53	11	0–15.2	Wang et al. 2019	G.L. Jacobson et al., unpub.
NE	Benson Pond, MA	40791	42.378	−73.1	776	19.1		57	11	0–14.5	Author submitted	[61]
NE	Berry Pond, MA	40793	42.62	−71.09	508	22.3	*	160	9	0–14.6	Author submitted	[61]
NE	Black Pond, MA	40795	41.328	−70.79	534	22.2		79	11	0–9.9	Author submitted	[61]
NE	Blaney's Pond, MA	40797	41.472	−70.77	534	22.2		71	12	0–13.6	Oswald et al. 2018	[61]
NE	Blood Pond, MA	40799	42.08	−71.96	631	21.5	*	143	15	0–14.9	Author submitted	[61]
NE	Deep Taunton Pond, MA	40844	41.882	−71.01	590	22.2		46	17	0.1–16.0	Author submitted	[61]
NE	Deep-Falmouth Pond, MA	40803	41.564	−70.64	561	21.8	*	146	15	0–15.4	Author submitted	[61]
NE	Doe Pond, MA	40854	42.175	−72.7	525	21.8		91	12	0–15.1	Author submitted	[61]
NE	Guilder Pond, MA	40875	42.109	−73.44	676	20.0		55	9	0–15.2	Author submitted	[61]
NE	Little Royalston Pond, MA	40988	42.676	−72.19	621	20.6		88	8	0–15.1	Author submitted	[61]
NE	North Pond, MA	1817	42.652	−73.05	785	18.9		55	13	0–13.3	Neotoma 1	[64,65]
NE	Uncle Seth's Pond, MA	41018	41.433	−70.67	536	21.9		60	7	0–13.4	Author submitted	[61]
NE	Ware Pond, MA	41025	42.482	−70.88	484	22.5		59	8	0–11.2	Author submitted	[61]
NE	Winneconnet Pond, MA	2959	41.967	−71.12	582	22.1		70	12	0–16.5	Wang et al. 2019	[66]
NE	Little Willey Pond, NH	40992	43.292	−71.18	554	20.3		73	6	0–13.4	Author submitted	[61]
NE	Ballston Lake, NY	15909	42.95	−73.85	483	21.4		59	6	1.7–12.5	Neotoma 1	[67]
NE	Balsam Lake, NY	15350	42.029	−74.6	788	18.6		89	7	0–18.2	Neotoma 2	[68]
NE	Sears Pond, NY	41001	40.886	−72.58	509	22.3		33	11	0–10.7	Author submitted	[61]
NE	Spruce Pond, NY	2593	41.237	−74.18	630	22.1	*	178	9	0–17.0	Blois et al. 2011	[69,70]
NE	Sutherland Pond, NY	2617	41.391	−74.04	551	22.6	*	183	10	0–14.2	This study	[69,71]
NE	Knob Hill Pond, VT	40914	44.36	−72.37	599	18.9	*	138	12	0–14.1	Author submitted	[61]
NE	Lac Colin, Quebec	1569	46.717	−70.3	828	16.6		45	8	0–12.3	Wang et al. 2019	[72]
NE	Lac Castor, Quebec	346	46.614	−73	678	18.2		48	6	1.8–10.8	Wang et al.	A.C. Larouche et al., unpub.

Supplementary Table 2. Climate anomalies relative to pre-industrial from the climate simulations. Anomalies are shown for the the Midwest, Great Lakes and Northeast regions at 1-ka intervals as shown in Fig. 4. Anomalies are shown for annual precipitation (Δ*P*), annual potential evapotranspiration (PET), for annual water balance (i.e., Δ(*P*−PET)), and July temperature (ΔT_{jul}). The lower numbers indicate percentage change vs. preindustrial for *P*, PET, and water balance. Note that the 12, 11 and 9 ka BP values are based on CESM1 simulations, while other periods represent the 100-year averages derived from MPI-ESM and EC-Earth (2M). The mean values for PI are given in addition as reference.

ka	Δ*P* (mm/a)			ΔPET (mm/a)			Δ(*P*−PET) (mm/a)			ΔT_{jul} (°C)			ΔGDD5 (°C days)		
	Mid-west	Great Lakes	North-east	Mid-west	Great Lakes	North-east	Mid-west	Great Lakes	North-east	Mid-west	Great Lakes	North-east	Mid-west	Great Lakes	North-east
12	−124	−182	−176	−181	−214	−227	+57	+32	+51	−2.4	−2.3	−2.9	−842	−1000	−1065
	−24%	−23%	−15%	−29%	−33%	−42%	+49%	+24%	+8%						
11	−114	−154	−174	−138	−154	−149	+24	−0	−25	−0.9	−0.7	−0.5	−612	−668	−649
	−22%	−20%	−15%	−22%	−24%	−28%	+21%	−0%	−4%						
9	−106	−125	−152	−73	−89	−79	−33	−36	−73	+1.1	+0.8	+1.5	−271	−298	−292
	−21%	−16%	−13%	−12%	−14%	−15%	−28%	−27%	−12%						
8	−131	−95	−63	+72	+34	+26	−203	−129	−89	+4.3	+2.5	+2.4	+471	+245	+189
	−23%	−11%	−6%	+14%	+6%	+5%	−435%	−47%	−15%						
7	−115	−90	−58	+59	+23	+20	−175	−114	−78	+3.4	+1.8	+1.8	+408	+199	+167
	−21%	−11%	−6%	+12%	+4%	+4%	−376%	−42%	−13%						
6	−91	−80	−33	+55	+24	+21	−146	−104	−54	+2.8	+1.4	+1.4	+313	+141	+119
	−16%	−10%	−3%	+11%	+4%	+5%	−312%	−38%	−9%						
5	−64	−56	−25	+39	+20	+16	−103	−76	−41	+1.8	+1.0	+0.9	+258	+140	+110
	−11%	−7%	−2%	+8%	+4%	+3%	−220%	−28%	−7%						
4	−30	−21	−18	+15	+1	+6	−45	−22	−24	+0.7	+0.1	+0.4	+116	+26	+41
	−5%	−3%	−2%	+3%	+0%	+1%	−95%	−8%	−4%						
3	−9	−13	−15	+13	+6	+8	−22	−19	−23	+0.5	+0.1	+0.2	+79	+36	+38
	−2%	−2%	−1%	+3%	+1%	+2%	−47%	−7%	−4%						
2	−9	−2	−11	+5	−3	+0	−14	+1	−11	+0.3	−0.2	+0.0	+17	−25	−16
	−2%	+0%	−1%	+1%	−1%	+0%	−31%	0%	−2%						
1	+5	−1	−5	+3	+3	+3	+2	−4	−8	+0.0	+0.0	+0.0	+6	+7	+12
	+1%	+0%	+0%	+1%	+1%	+1%	+4%	−2%	−1%						
PI 2M	559 mm	832 mm	1052 mm	512 mm	559 mm	469 mm	47 mm	273 mm	583 mm	20.6 °C days	20.8 °C days	18.8 °C days	2077 °C days	2223 °C days	1808 °C days
PI CESM1	512 mm	788 mm	1159 mm	628 mm	652 mm	535 mm	−116 mm	136 mm	624 mm	27.6 °C days	25.9 °C days	22.4 °C days	2812 °C days	2849 °C days	2221 °C days

Supplementary Table 3. Main periodicities (ka) observed in the wavelet analyses. Periodicities are shown for the Water balance and July temperature anomalies of the paleoclimate reconstructions and the transient model simulations for the Midwest and Northeast regions, using the AR1 method to describe the background variability. The percentages in parentheses indicate how often a given periodicity was significant in the six different red-noise based methods tested (AR-1; ARIMA(101), ARIMA(102), ARIMA(103), ARIMA(104) and ARIMA(105)). The periodicities marked in *red* were significant in three or fewer of the six methods and have been filtered out of the results shown in Fig. 6.

Region	Variable	Fossil site / simulation	First	Second	Third	Fourth	Fifth
Midwest	Water balance	Sharkey Lake, MN	0.696 (100%)	1.171 (83 %)	NA	NA	NA
Midwest	Water balance	Kimble Pond, MN	0.214 (17 %)	NA	NA	NA	NA
Midwest	Water balance	Steel Lake, MN	0.325 (100%)	0.238 (50 %)	0.773 (67 %)	NA	NA
Midwest	Water balance	Kettle Lake, ND	0.429 (50 %)	NA	NA	NA	NA
Midwest	Water balance	Moon Lake, ND	0.510 (100%)	NA	NA	NA	NA
Midwest	Water balance	Jones Lake, Manitoba	0.314 (100%)	NA	NA	NA	NA
Northeast	Water balance	Berry Pond, MA	0.214 (100%)	0.429 (100%)	NA	NA	NA
Northeast	Water balance	Blood Pond, MA	0.857 (100%)	0.386 (83 %)	NA	NA	NA
Northeast	Water balance	Deep-Falmouth Pond, MA	0.444 (83 %)	0.606 (100%)	NA	NA	NA
Northeast	Water balance	Spruce Pond, NY	0.510 (100%)	NA	NA	NA	NA
Northeast	Water balance	Sutherland Pond, NY	0.273 (100%)	0.510 (100%)	NA	NA	NA
Northeast	Water balance	Knob Hill Pond, VT	0.293 (100%)	NA	NA	NA	NA
Midwest	Water balance	TraCE-21ka	0.175 (100%)	0.104 (100%)	0.034 (100%)	0.020 (83 %)	NA
Midwest	Water balance	EC-Earth	0.142 (100%)	0.175 (100%)	0.074 (100%)	0.034 (100%)	0.020 (67 %)
Midwest	Water balance	MPI-ESM	0.274 (100%)	0.163 (100%)	0.124 (100%)	0.033 (100%)	0.020 (67 %)
Northeast	Water balance	TraCE-21ka	0.163 (100%)	0.034 (100%)	NA	NA	NA
Northeast	Water balance	EC-Earth	0.169 (100%)	0.104 (100%)	NA	NA	NA
Northeast	Water balance	MPI-ESM	0.274 (100%)	0.124 (100%)	0.187 (100%)	0.033 (100%)	NA
Midwest	July temperature	Sharkey Lake, MN	0.146 (17 %)	0.800 (100%)	NA	NA	NA
Midwest	July temperature	Kimble Pond, MN	0.255 (83 %)	NA	NA	NA	NA
Midwest	July temperature	Steel Lake, MN	0.336 (83 %)	0.246 (17 %)	NA	NA	NA
Midwest	July temperature	Kettle Lake, ND	0.187 (17 %)	0.303 (17 %)	NA	NA	NA
Midwest	July temperature	Moon Lake, ND	0.510 (67 %)	NA	NA	NA	NA
Midwest	July temperature	Jones Lake, Manitoba	0.546 (100%)	0.200 (17 %)	NA	NA	NA
Northeast	July temperature	Berry Pond, MA	0.429 (100%)	0.606 (100%)	NA	NA	NA
Northeast	July temperature	Blood Pond, MA	0.800 (100%)	0.566 (100%)	NA	NA	NA
Northeast	July temperature	Deep-Falmouth Pond, MA	0.303 (100%)	0.528 (100%)	NA	NA	NA
Northeast	July temperature	Spruce Pond, NY	0.696 (100%)	0.193 (17 %)	NA	NA	NA
Northeast	July temperature	Sutherland Pond, NY	0.386 (100%)	NA	NA	NA	NA
Northeast	July temperature	Knob Hill Pond, VT	0.429 (100%)	NA	NA	NA	NA
Midwest	July temperature	TraCE-21ka	0.231 (100%)	0.294 (100%)	0.115 (100%)	0.034 (83 %)	NA
Midwest	July temperature	EC-Earth	0.142 (100%)	0.034 (100%)	NA	NA	NA
Midwest	July temperature	MPI-ESM	0.175 (100%)	0.274 (100%)	0.082 (100%)	0.034 (100%)	0.020 (100%)
Northeast	July temperature	TraCE-21ka	0.115 (100%)	NA	NA	NA	NA
Northeast	July temperature	EC-Earth	0.147 (100%)	0.034 (100%)	NA	NA	NA
Northeast	July temperature	MPI-ESM	0.175 (100%)	0.082 (100%)	0.033 (100%)	0.020 (100%)	0.015 (100%)

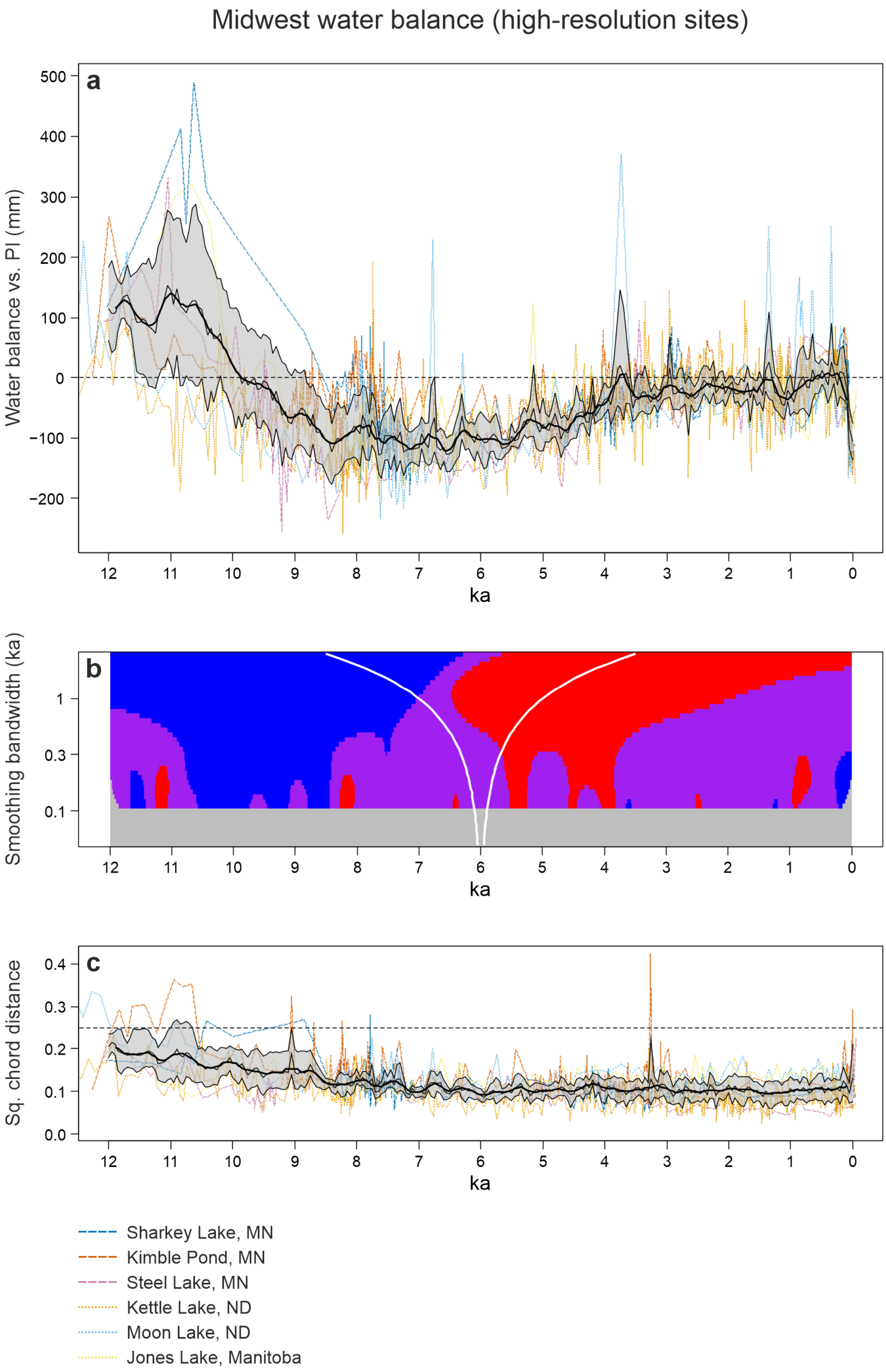

Supplementary Figure 1. Midwest water balance reconstructed with the boosted regression tree calibration model, from high-resolution sites (indicated in Supplementary Table 1) only. Panel **a** shows the reconstructed values from all fossil samples, with *colored lines* indicating the individual fossil sites. The *thin black line* shows the mean of all reconstructions interpolated at 50-year time step and the *thick black line* the five-point running mean. The *grey band* represent the 95% errors of the ensemble mean, calculated using 1000 bootstrap samples of all fossil datasets. The anomalies are expressed relative to the preindustrial period (0.25–0.75 ka). Panel **b** shows the SiZer map, indicating the significant features of the ensemble mean curve when smoothed at a range of bandwidths. For interpretation of the SiZer map, see caption to Fig. 2. Panel **c** shows the modern analog quality for the fossil pollen samples (squared chord distance to the best-matching calibration pollen sample). The 0.25 threshold for a good analog[73] is indicated with a *dashed line*. A mean curve with 95% errors is fitted to the pollen sample modern analog distances using an identical method with the paleoclimate reconstructions (panel **a**).

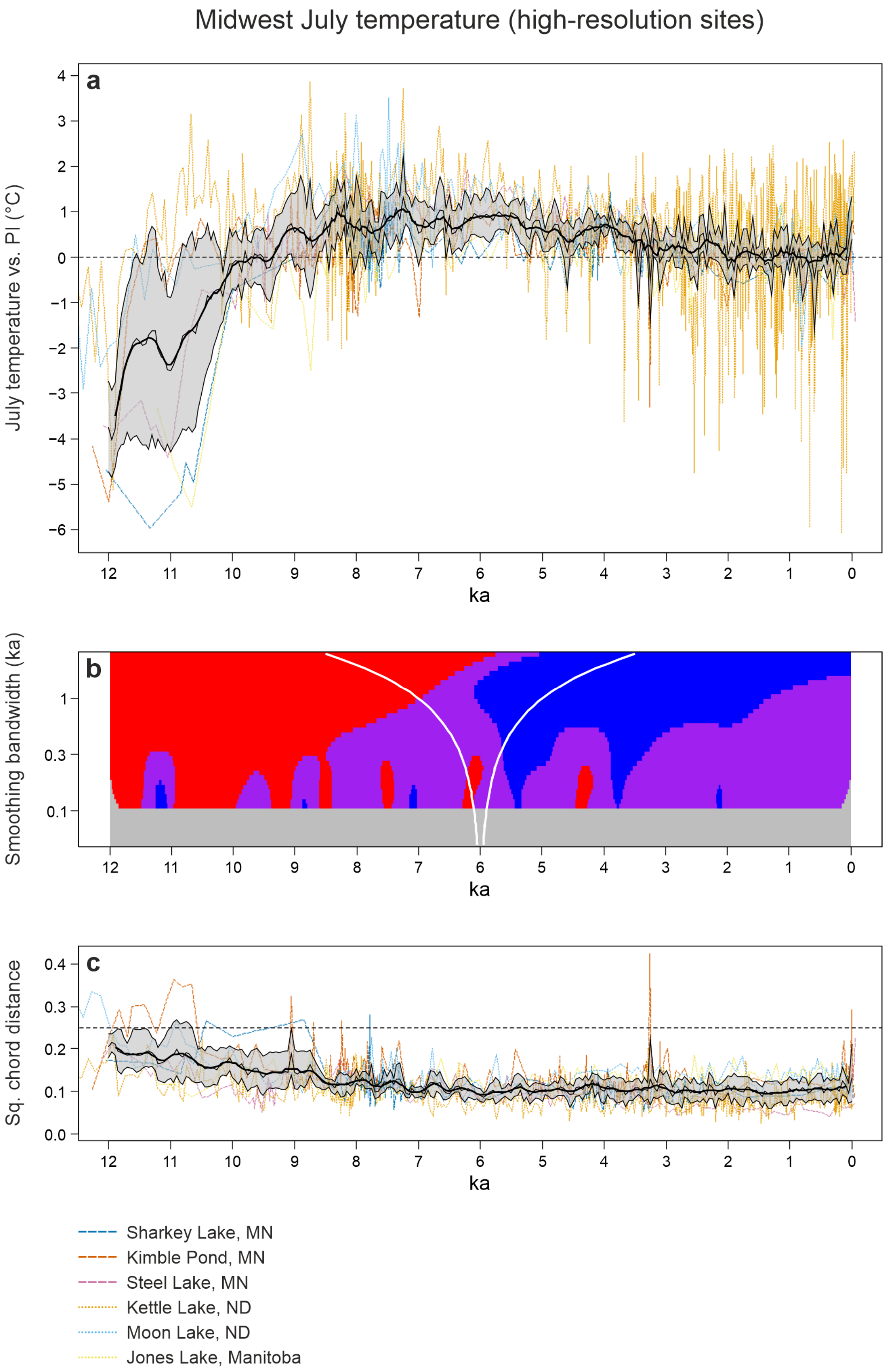

Supplementary Figure 2. As Supplementary Figure 1, but for Midwest July temperature (with high-resolution sites only).

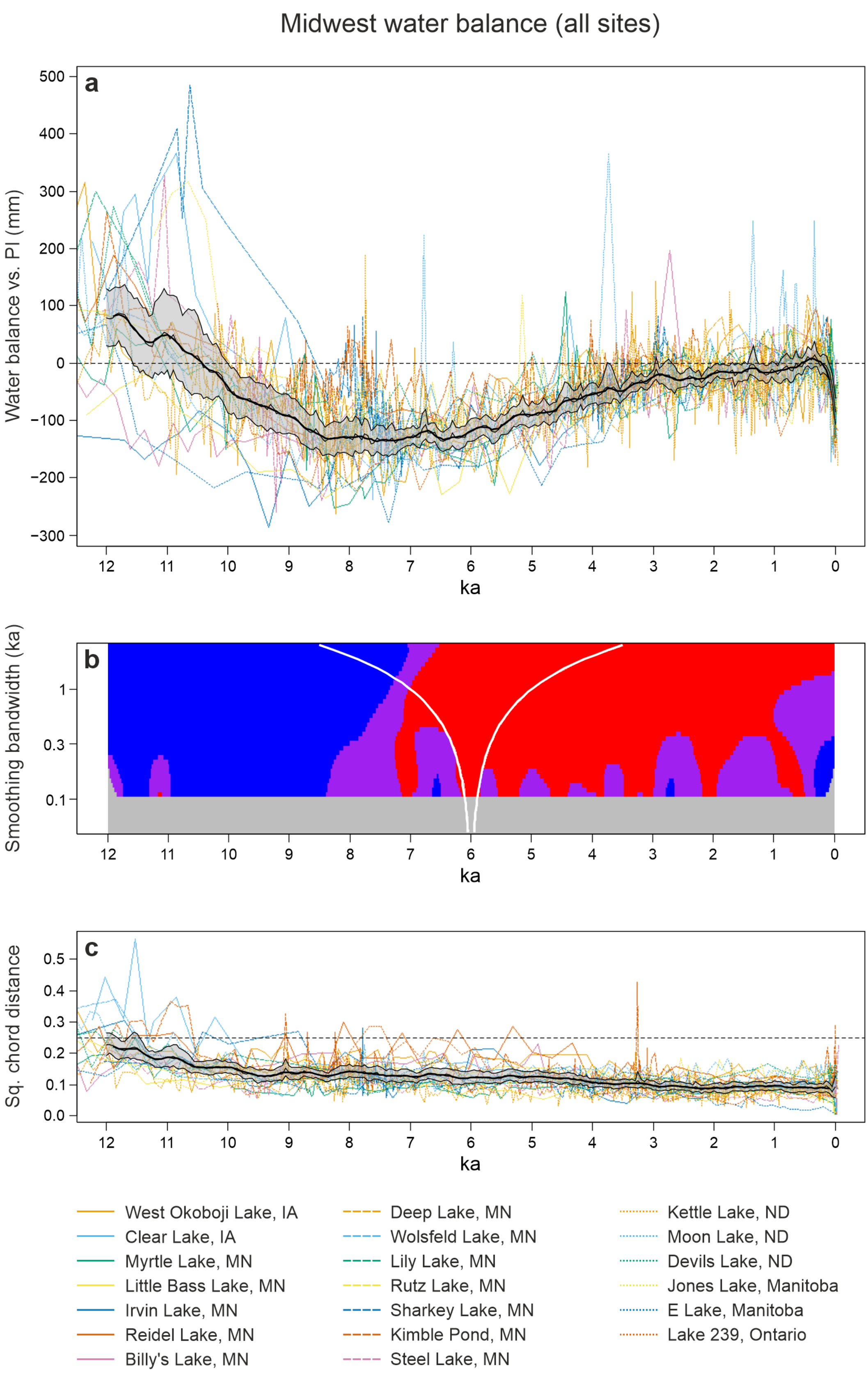

Supplementary Figure 3. As Supplementary Figure 1, but for Midwest water balance (with all sites).

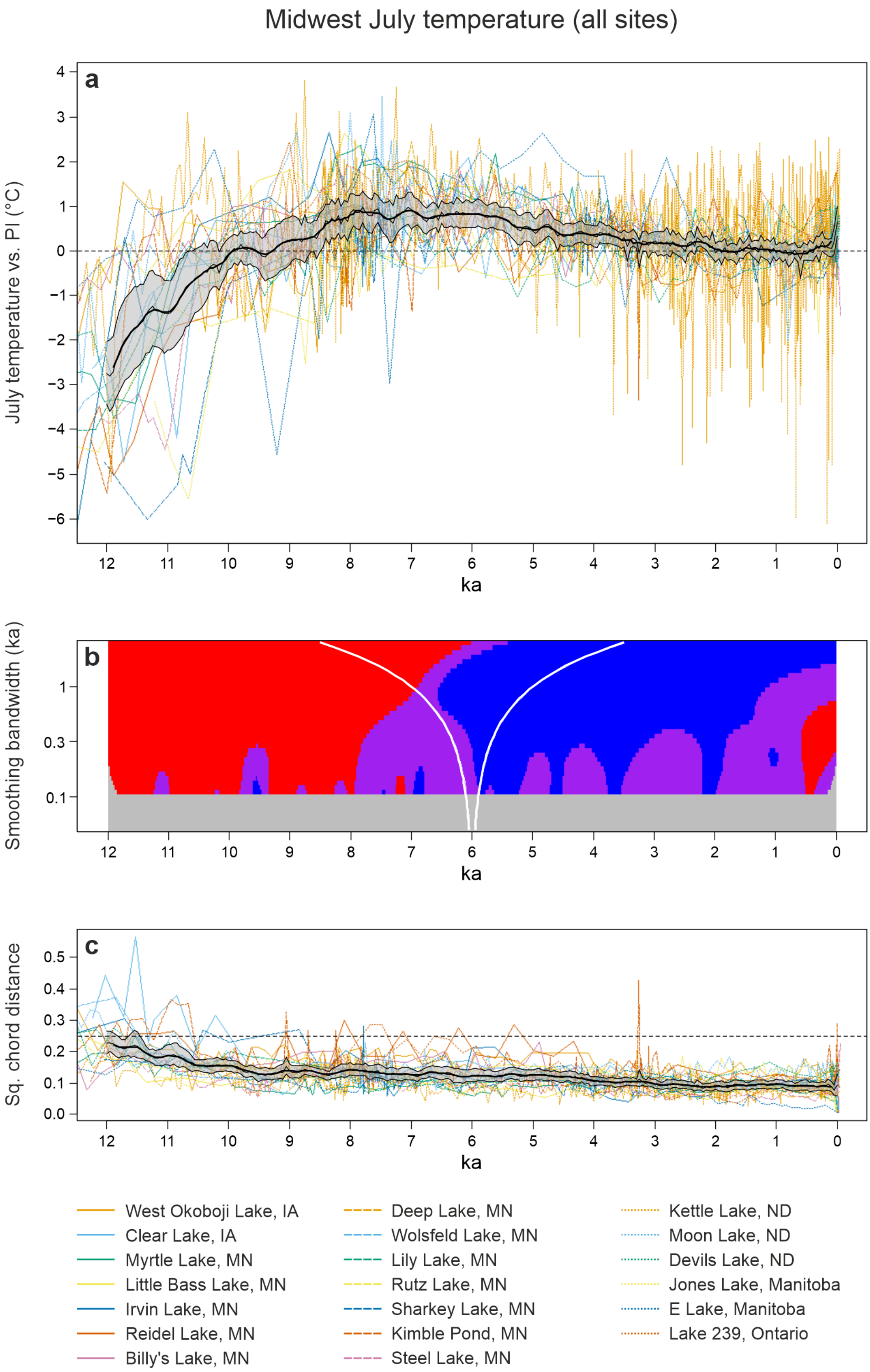

Supplementary Figure 4. As Supplementary Figure 1, but for Midwest July temperature (with all sites).

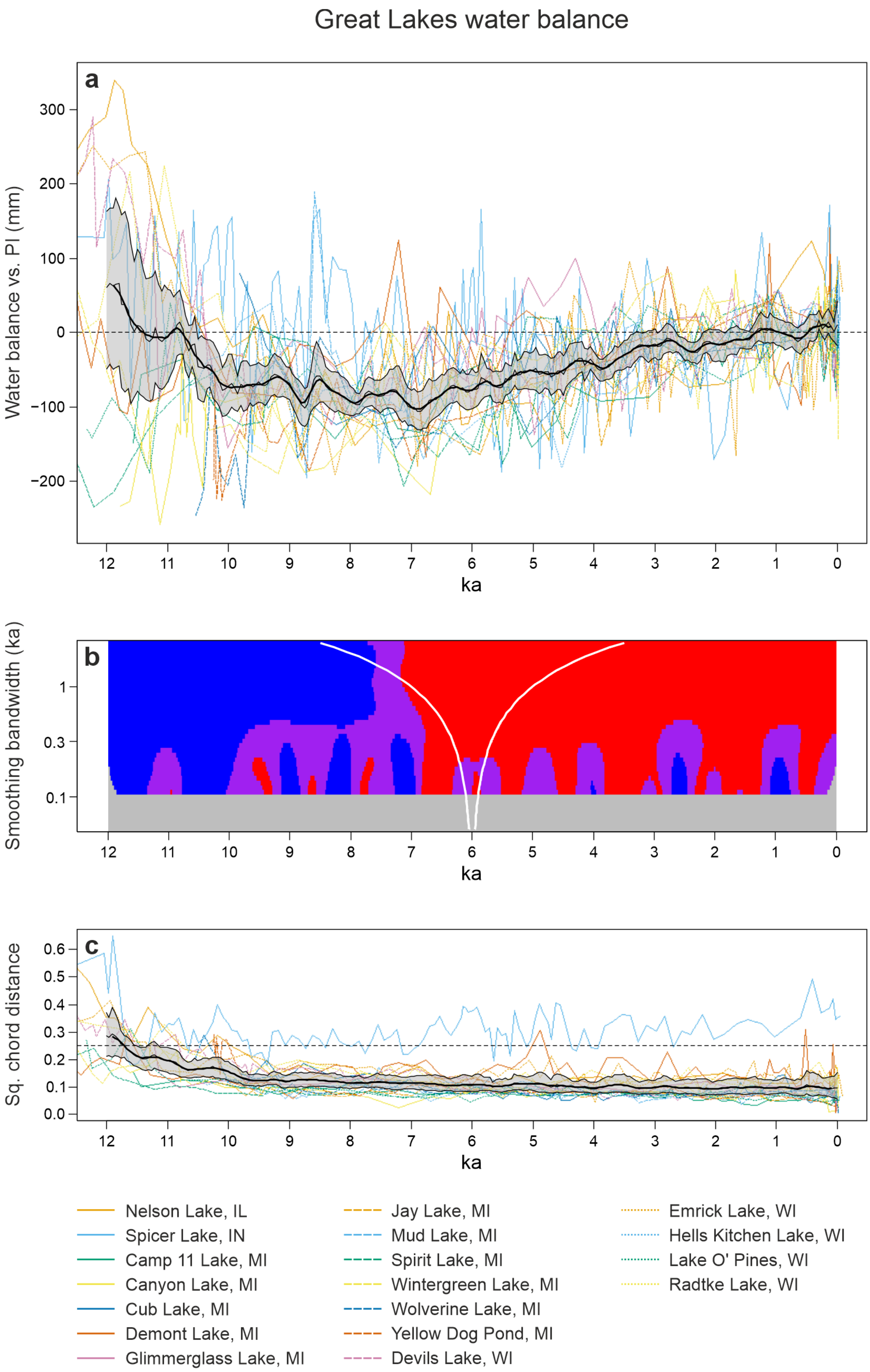

Supplementary Figure 5. As Supplementary Figure 1, but for Great Lakes water balance.

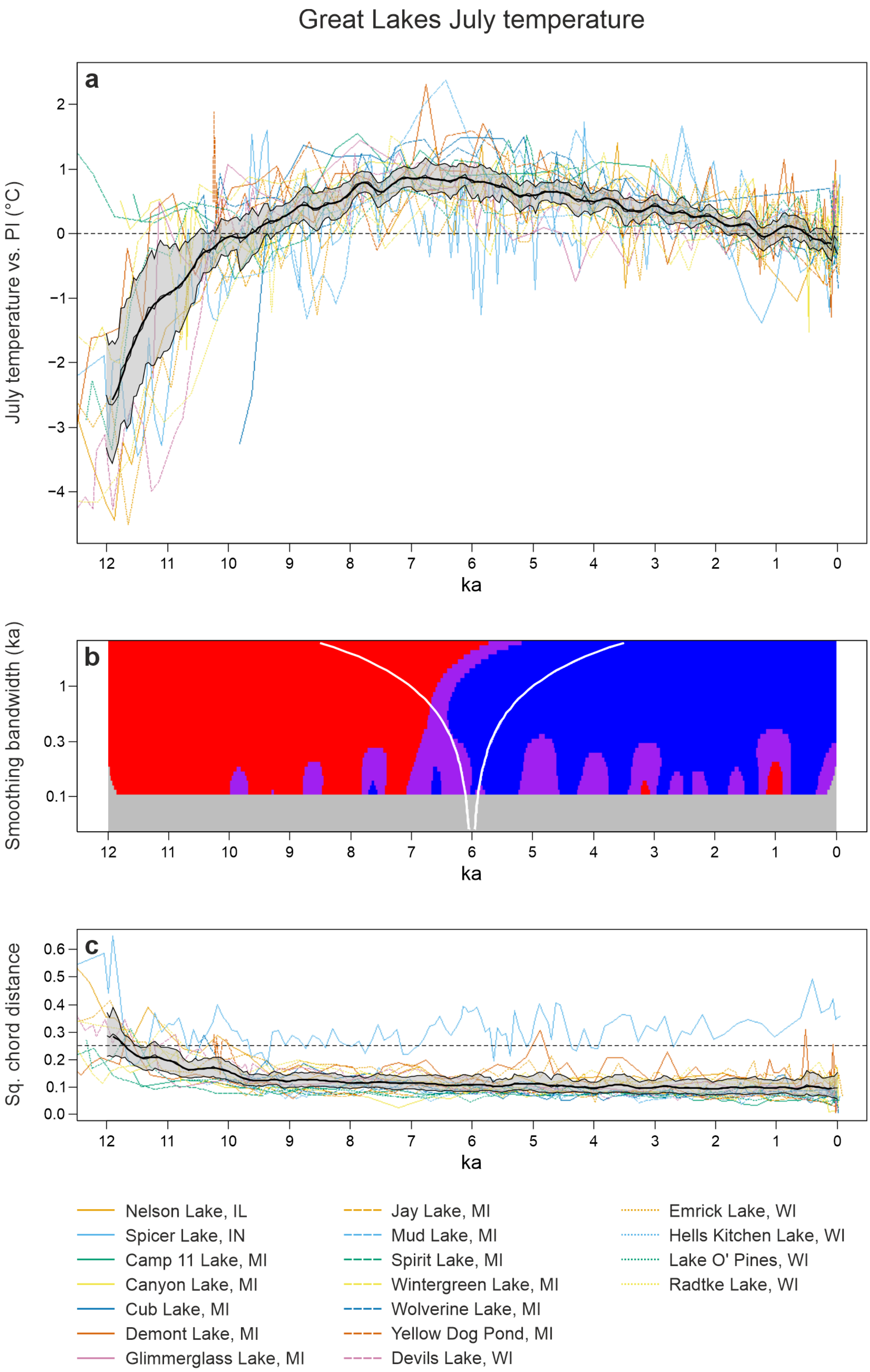

Supplementary Figure 6. As Supplementary Figure 1, but for Great Lakes July temperature.

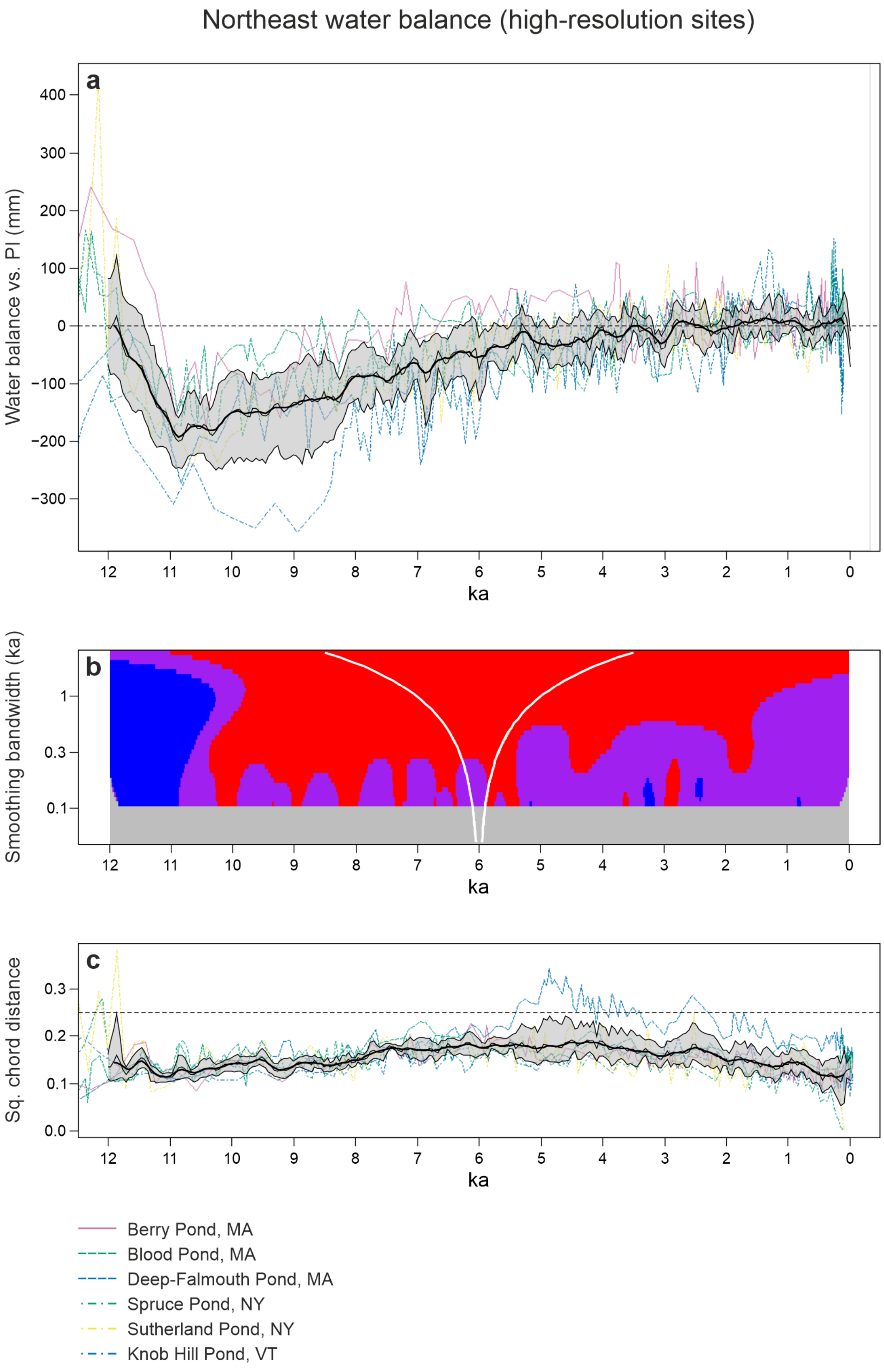

Supplementary Figure 7. As Supplementary Figure 1, but for Northeast water balance (high-resolution sites only).

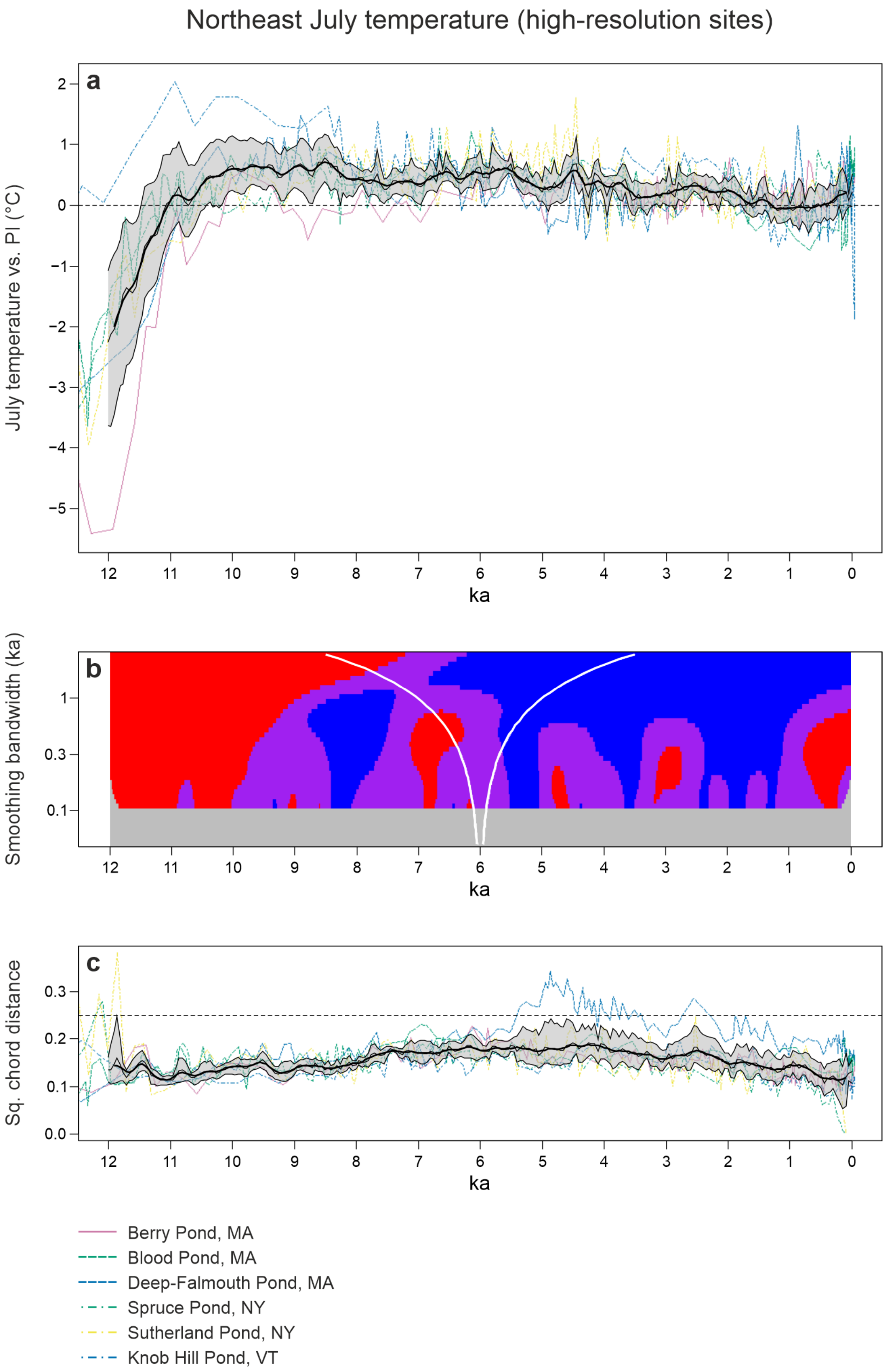

Supplementary Figure 8. As Supplementary Figure 1, but for Northeast July temperature (high-resolution sites only).

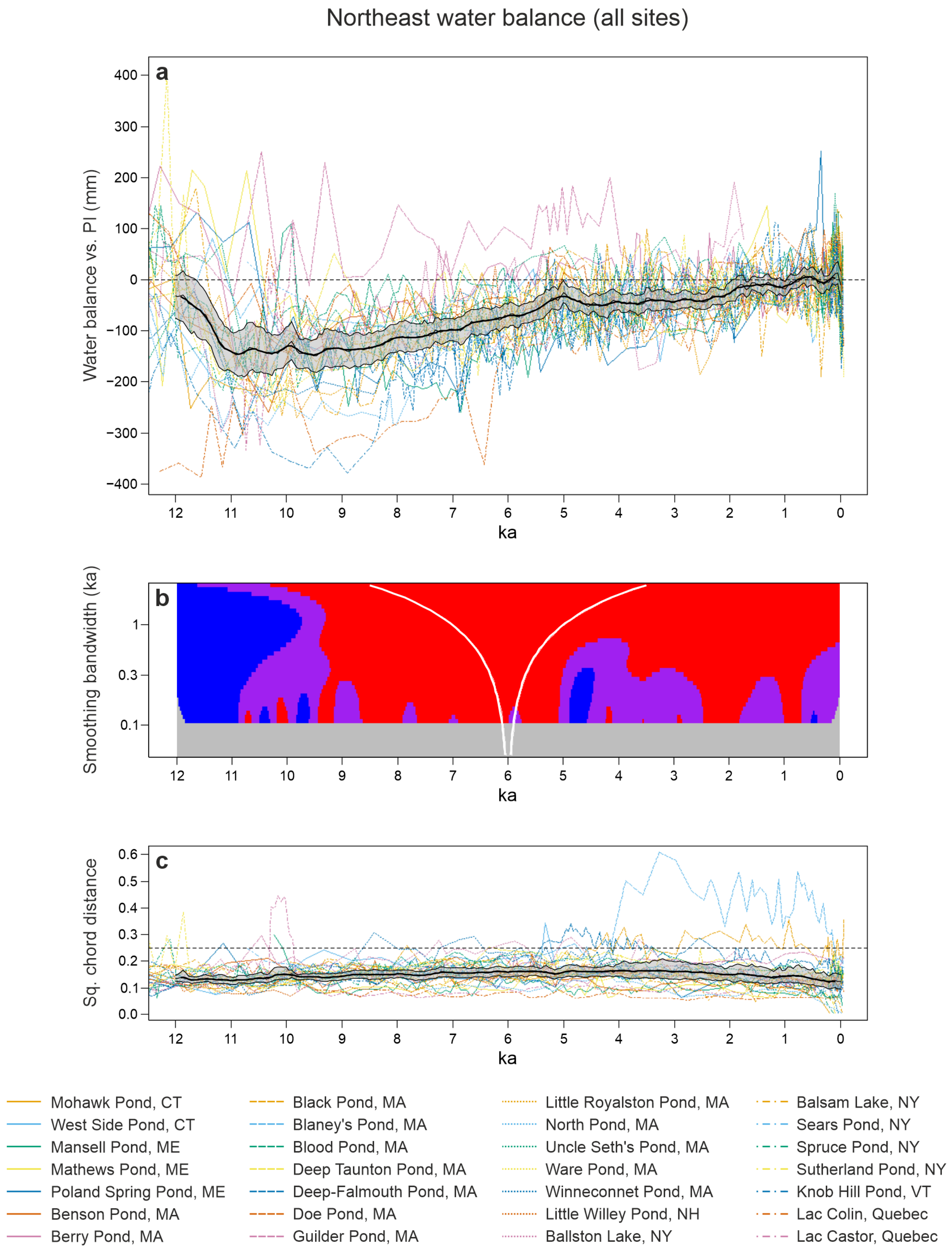

Supplementary Figure 9. As Supplementary Figure 1, but for Northeast water balance (all sites).

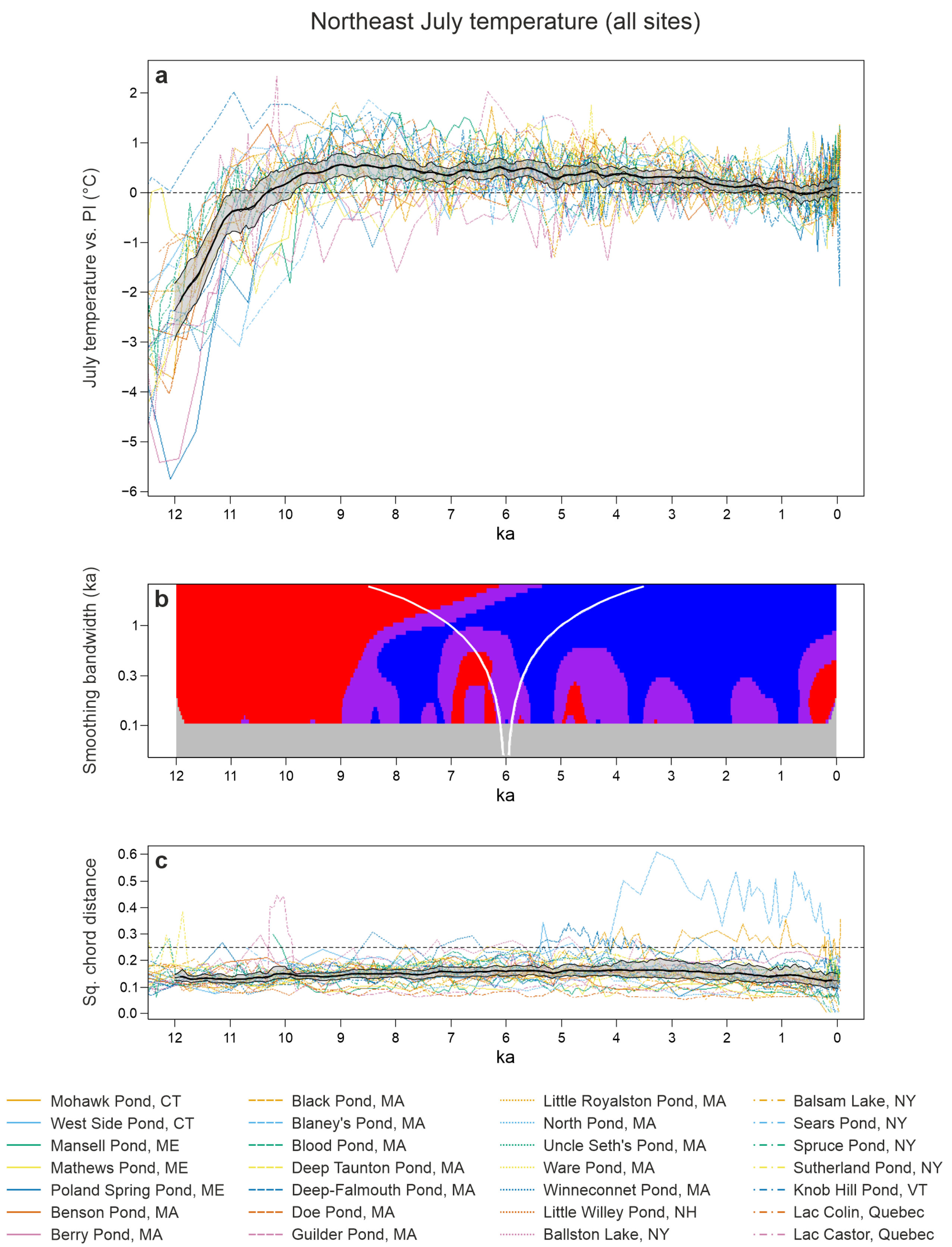

Supplementary Figure 10. As Supplementary Figure 1, but for Northeast July temperature (all sites).

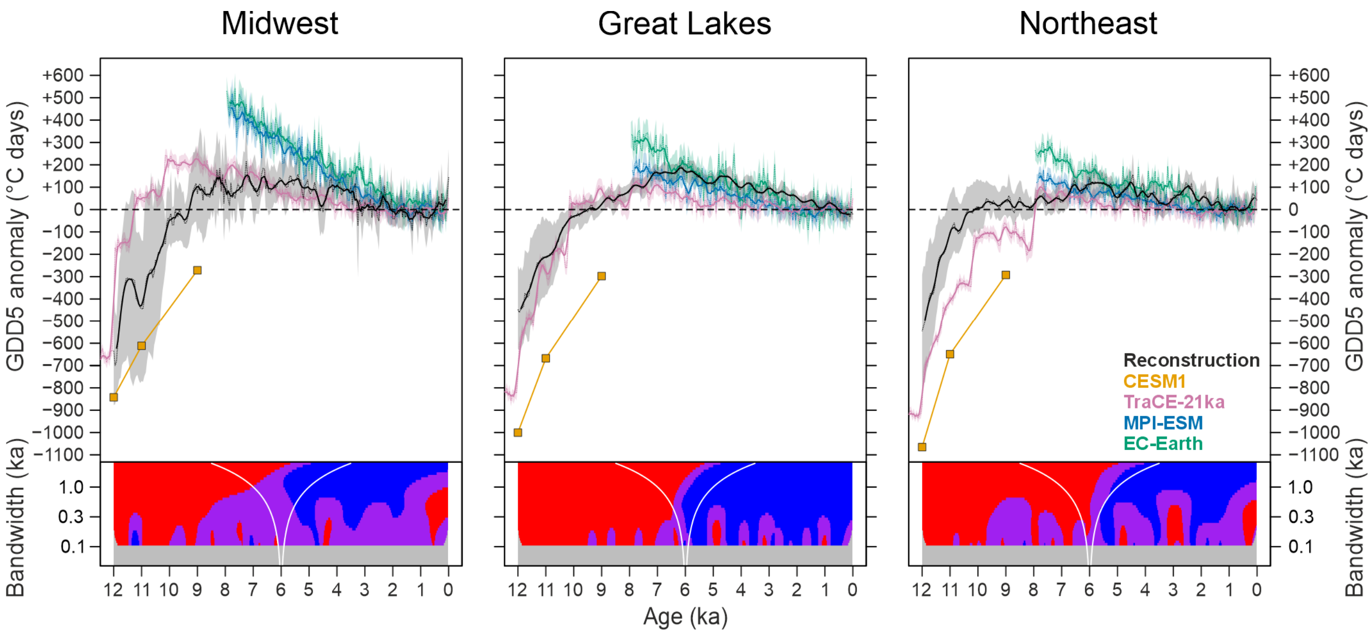

Supplementary Figure 11. As Fig. 2, but for growing degree days above 5ºC (GDD5).

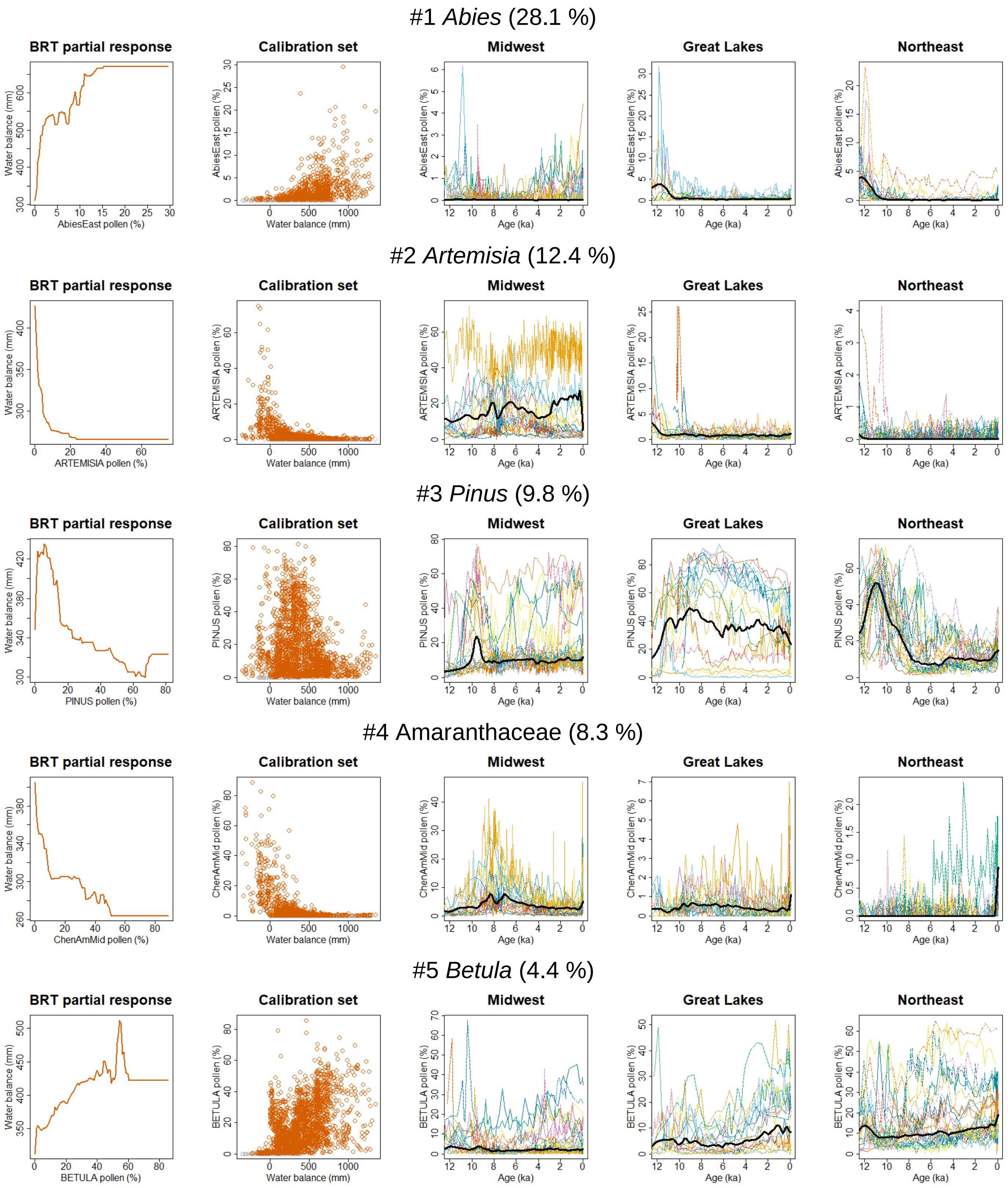

Supplementary Figure 12. Diagnostic plots for the ten most important predictor taxa in the pollen–water balance boosted regression tree (BRT) model. The taxa are listed from top to bottom according to decreasing percentage contribution (indicated in parentheses) to the BRT model. For each taxon we show the partial dependence plot[74] of the taxon in the BRT model, the climate response of the taxon in the calibration data set, and the fossil percentages of the taxon in the Midwest, Great Lakes and Northeast fossil data clusters (*colored lines* indicate individual fossil datasets following the symbology of Supplementary Figures 1–10 and the *thick black line* a LOWESS smoother (span 0.05) fitted to all datasets). *(Figure continues next page.)*

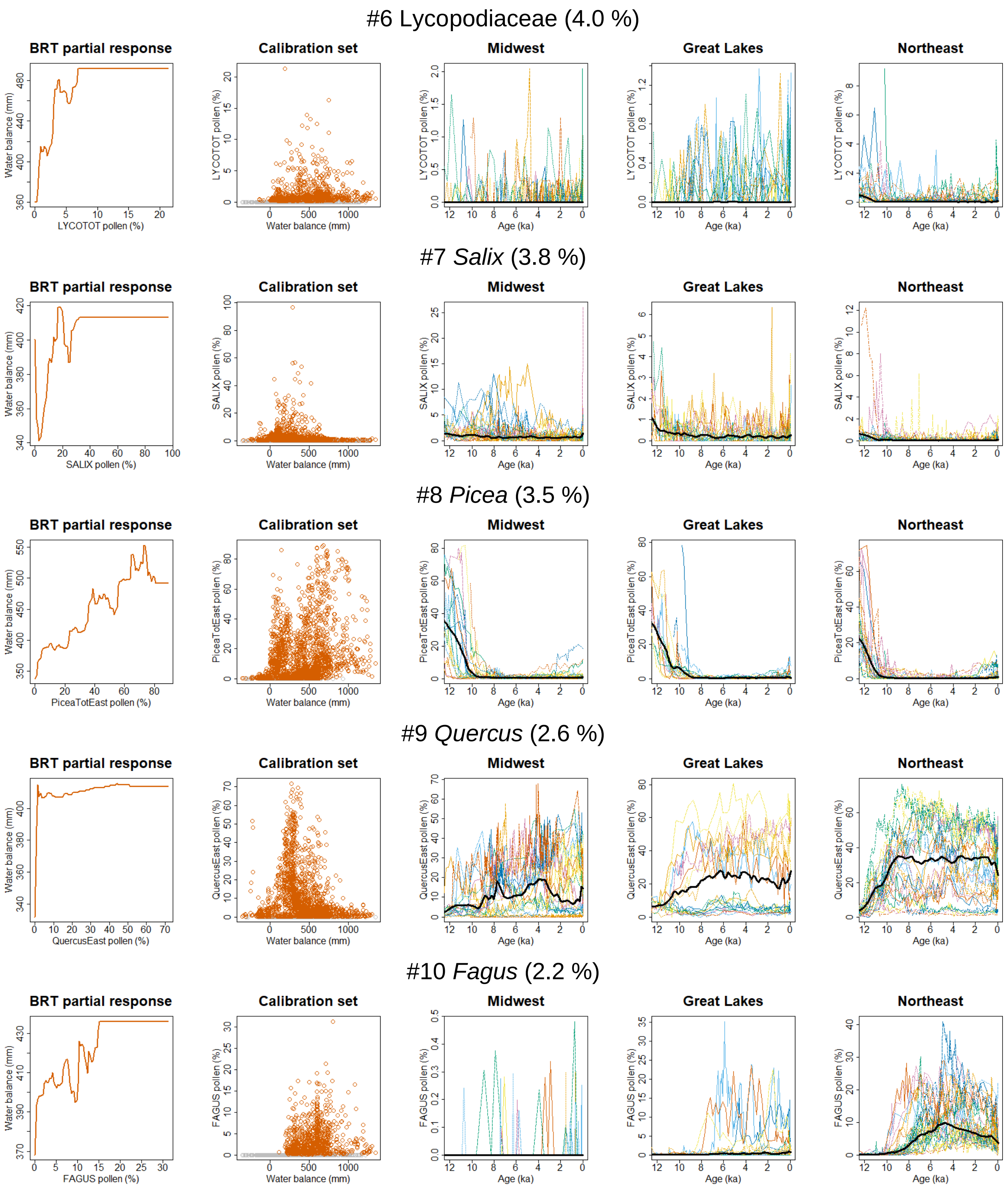

Supplementary Figure 12. *(continued)*

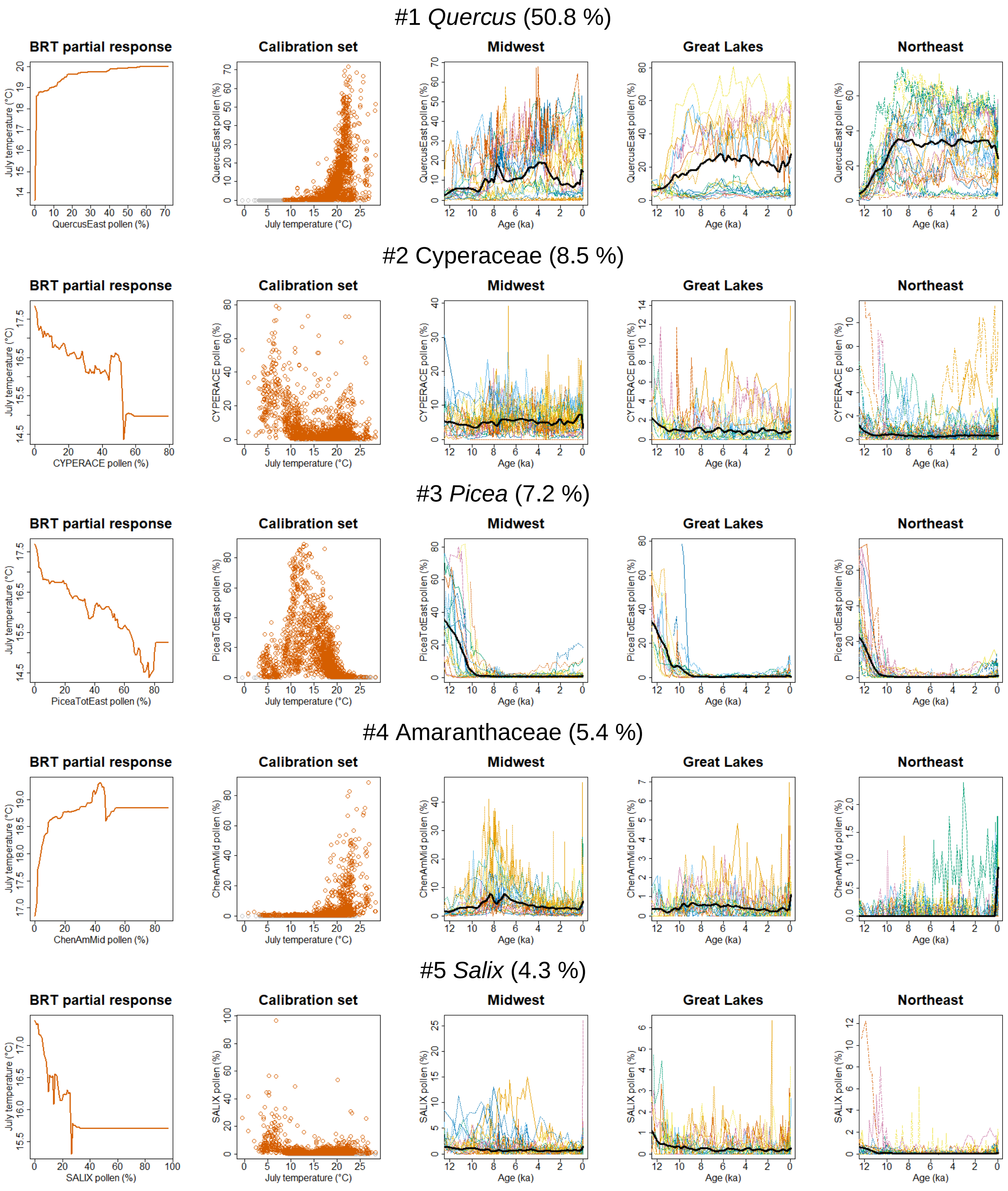

Supplementary Figure 13. Diagnostic plots for the ten most important predictor taxa in the pollen–July temperature boosted regression tree model. For further details see caption to Supplementary Figure 12. *(Figure continues on next page.)*

#6 *Betula* (3.8 %)

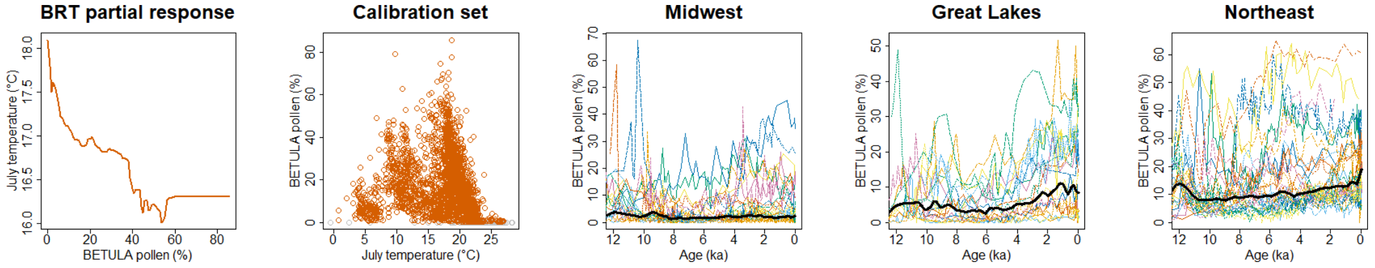

#7 *Pinus* (3.4 %)

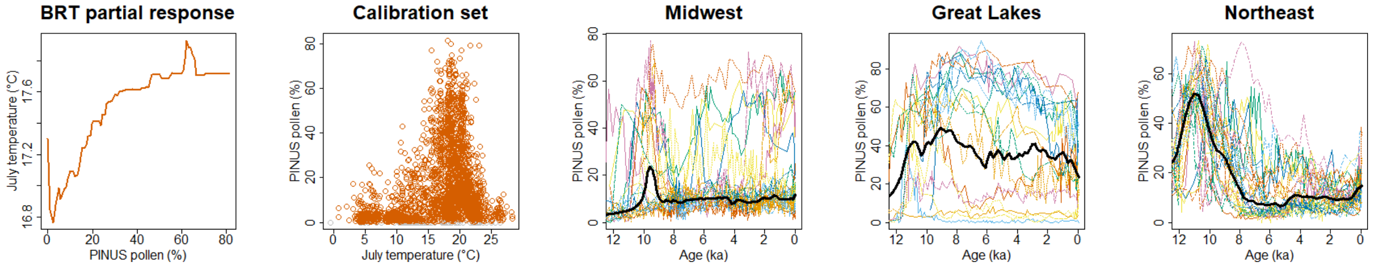

#8 *Alnus* (1.9 %)

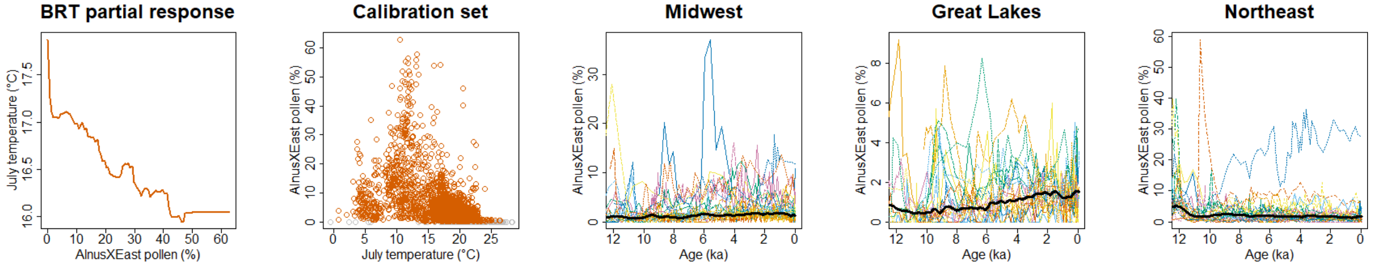

#9 Ericaceae (1.6 %)

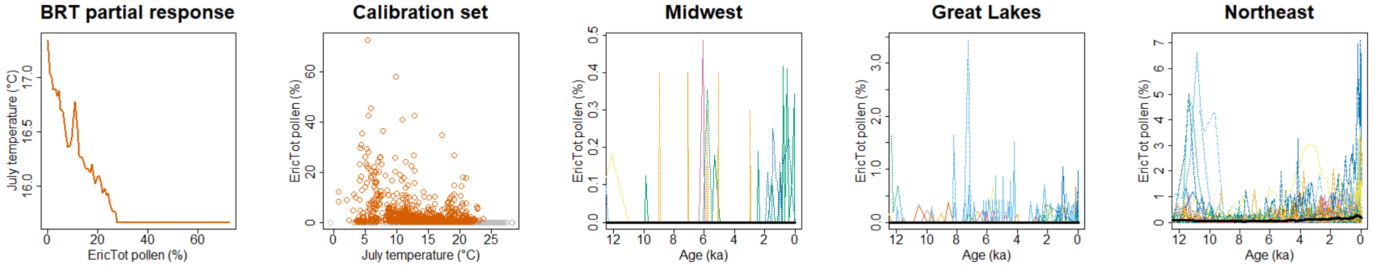

#10 *Oxyria* (1.6 %)

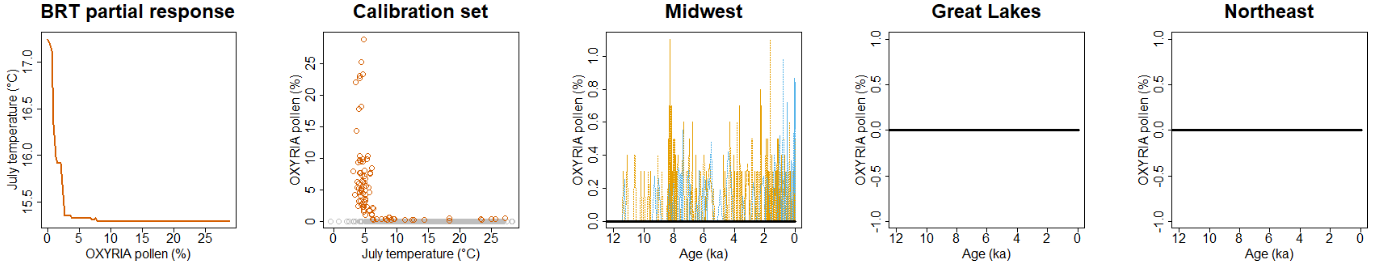

Supplementary Figure 13. *(continued)*

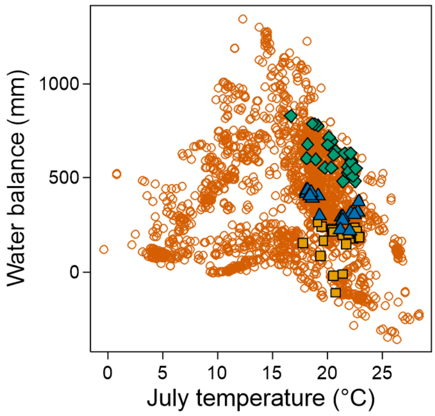

Supplementary Figure 14. Modern water balance and July temperature values for the pollen calibration samples and the fossil pollen sites. The pollen–climate calibration dataset sites are indicated with *empty circles* while the *filled symbols* indicate the fossil data sites from the three spatial clusters (*squares* = Midwest, *triangles* = Great Lakes, *diamonds* = Northeast).

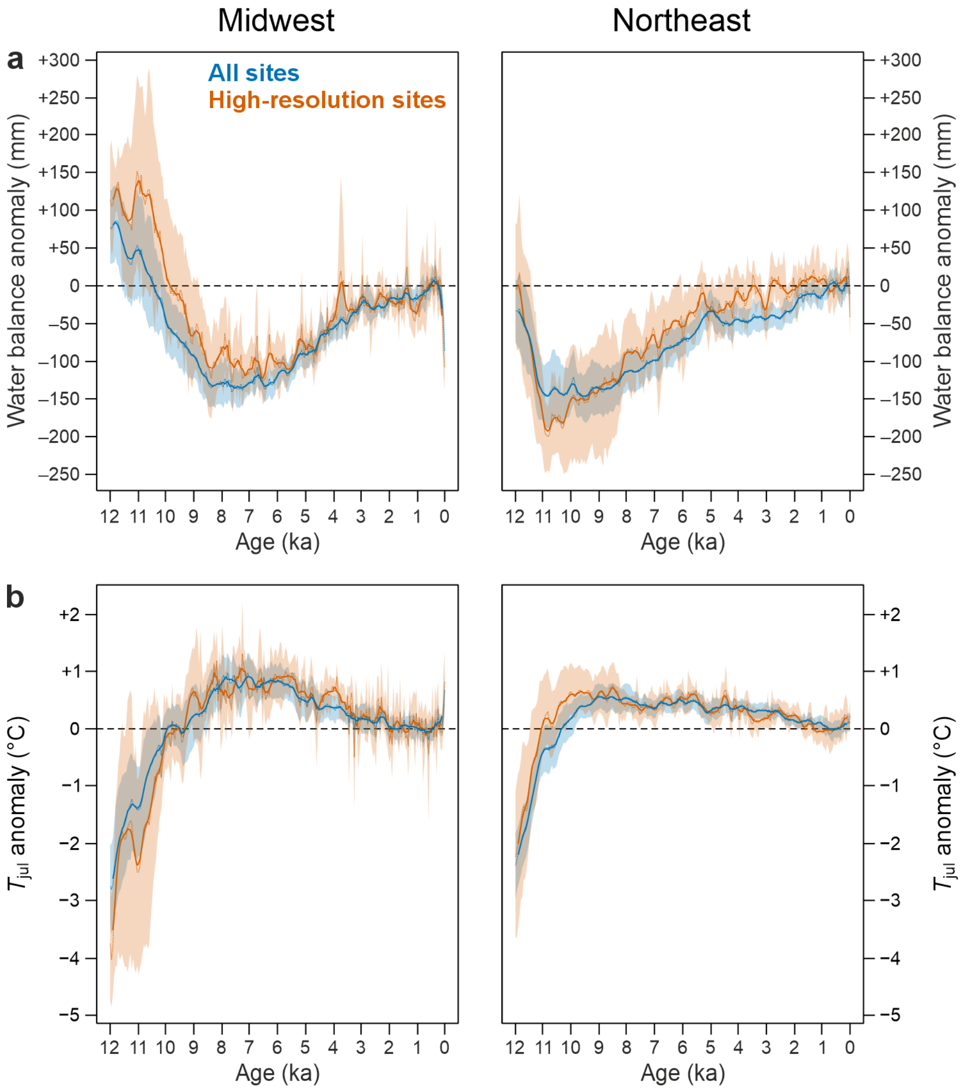

Supplementary Figure 15. Comparison of paleoclimate reconstructions for the Midwest and Northeast regions using all sites vs. subsets of high-resolution sites (see Supplementary Table 1). The comparison is shown for (**a**) annual water balance and (**b**) July mean temperature (T_{jul}) reconstructions. Symbology as in Fig. 2.

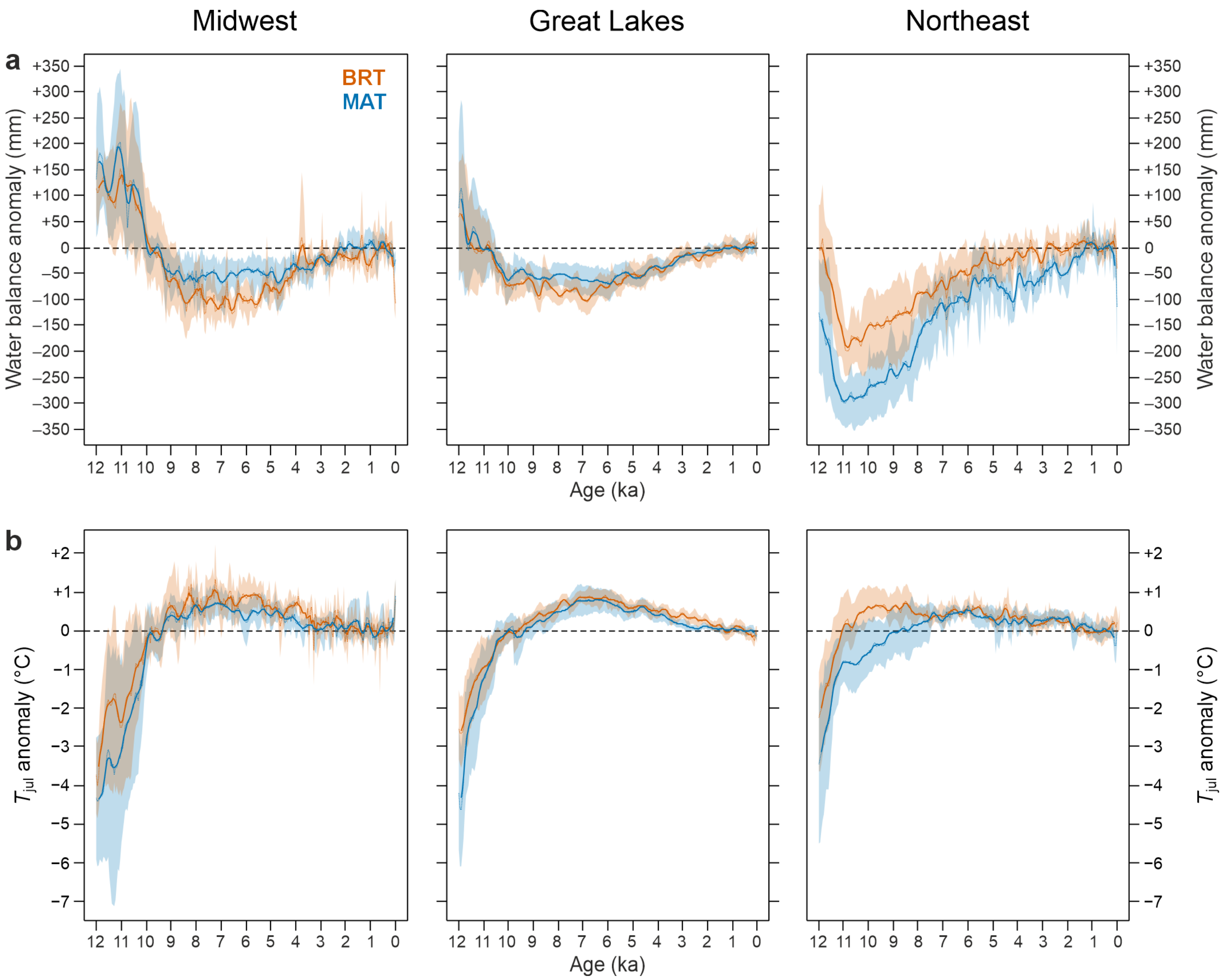

Supplementary Figure 16. Comparison of reconstructions with boosted regression tree (BRT) and the modern-analog technique (MAT) based pollen–climate calibration models. The comparison is shown for (**a**) annual water balance and (**b**) July mean temperature (T_{jul}) reconstructions in the three regions (Midwest, Great Lakes, and Northeast). Symbology as in Fig. 2.

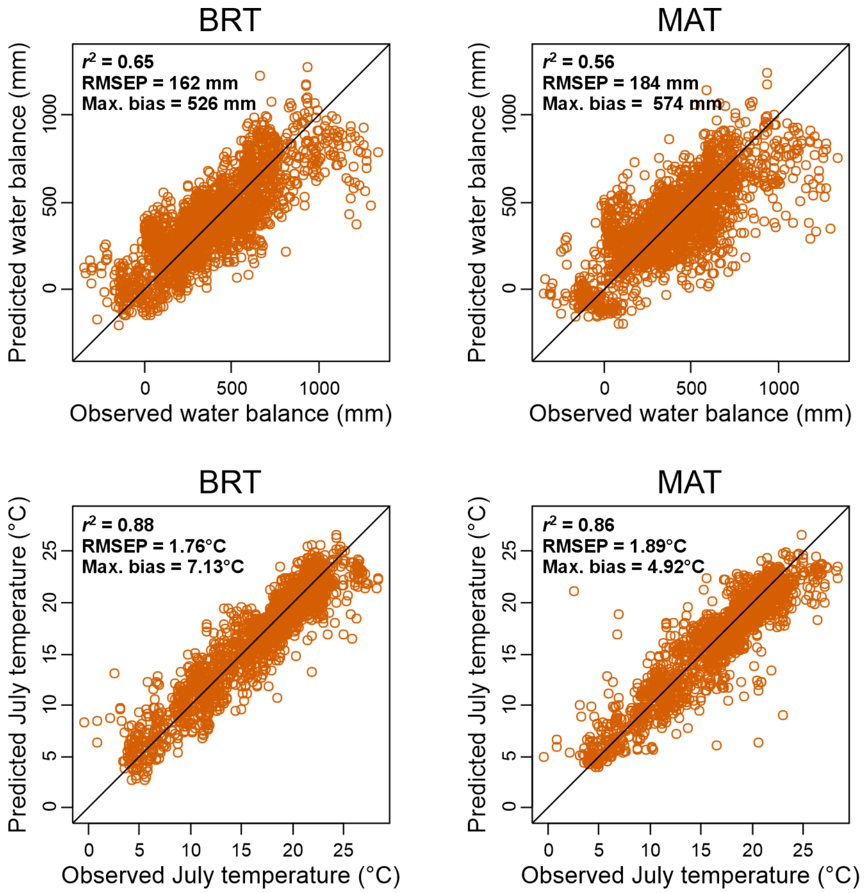

Supplementary Figure 17. Cross-validation performance of the pollen–climate calibration models**.** Models were prepared for reconstruction of two climate variables, water balance and July temperature, using two different approaches: boosted regression trees (BRT) and the modern-analog technique (MAT). The plots show the predicted vs. observed climate values for each model in *h*-block cross-validation[75]. The *h* used (500 km for water balance and 600 km for July temperature) was determined based on the range of a circular variogram fitted to the residuals of a weighted-averaging calibration model[75]. The cross-validation performance is summarized with three figures: coefficient of determination (r^2), the root-mean-square error of prediction (RMSEP), representing a typical prediction error for all samples, and the maximum (max.) bias, calculated as the largest mean of prediction residuals found for any of the 10 equal-length segments of the calibration data climate gradient and representing a “worst case” prediction error for a specific environment.

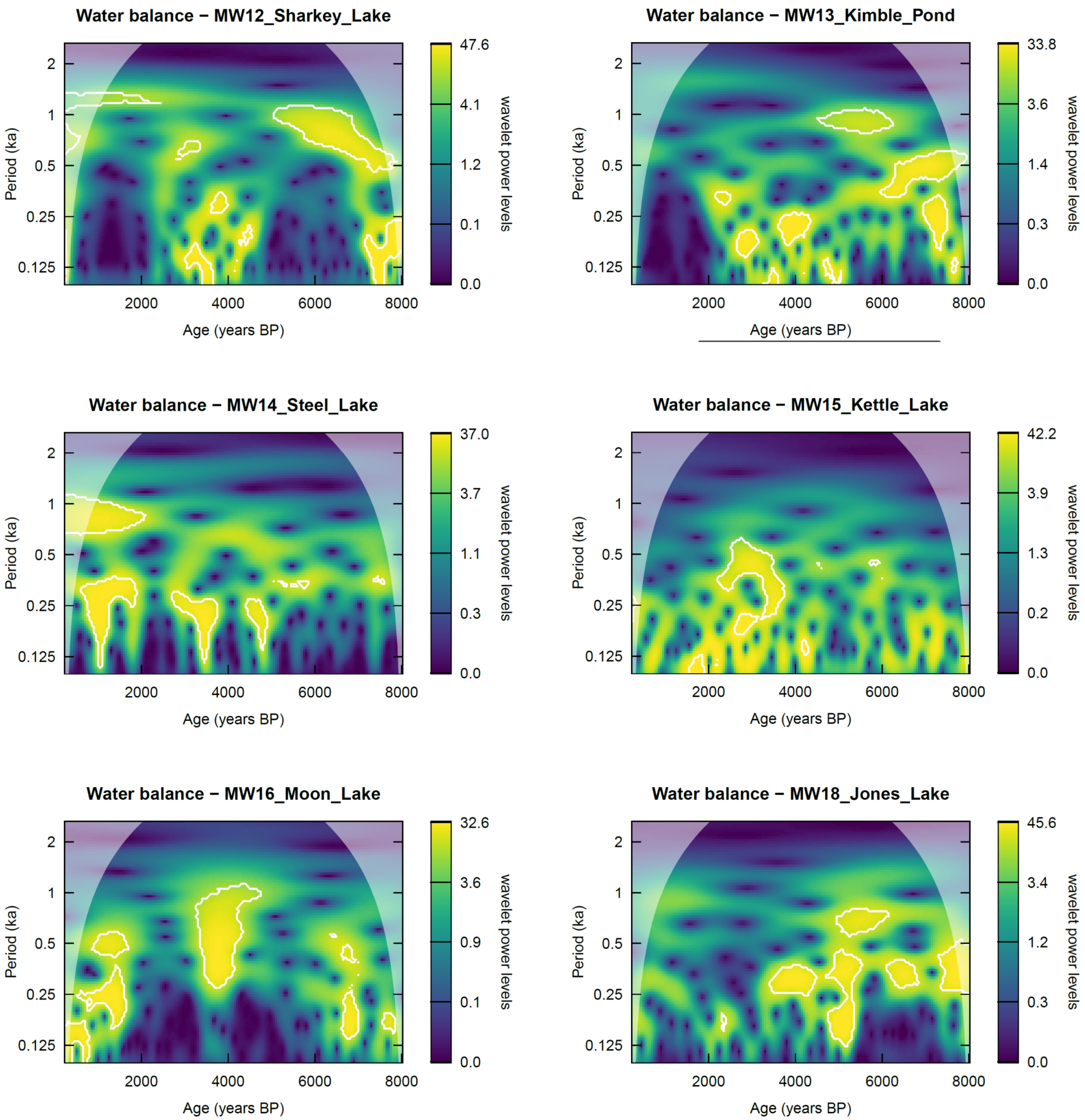

Supplementary Figure 18. Wavelet power spectra of the annual water balance reconstructions from the high-resolution fossil pollen sequences of the Midwest site cluster. Marked regions on the wavelet spectrum indicate significant power to a 95% confidence interval. The areas under the cone of influence show where edge effects are important. For a list of significant periodicities, see Supplementary Table 3.

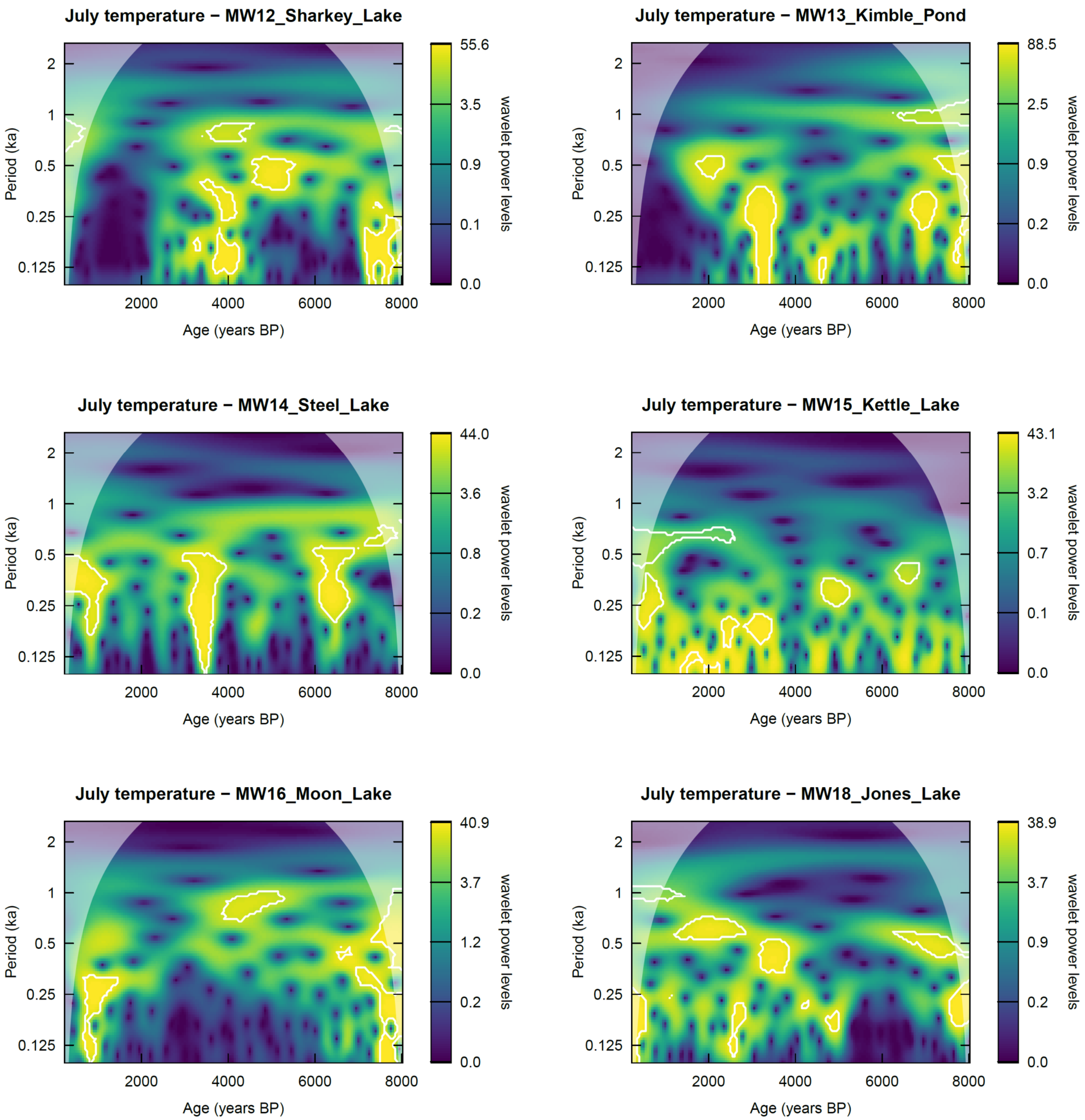

Supplementary Figure 19. As Supplementary Figure 18, but for July temperature reconstructions from the high-resolution fossil pollen sequences of the Midwest site cluster.

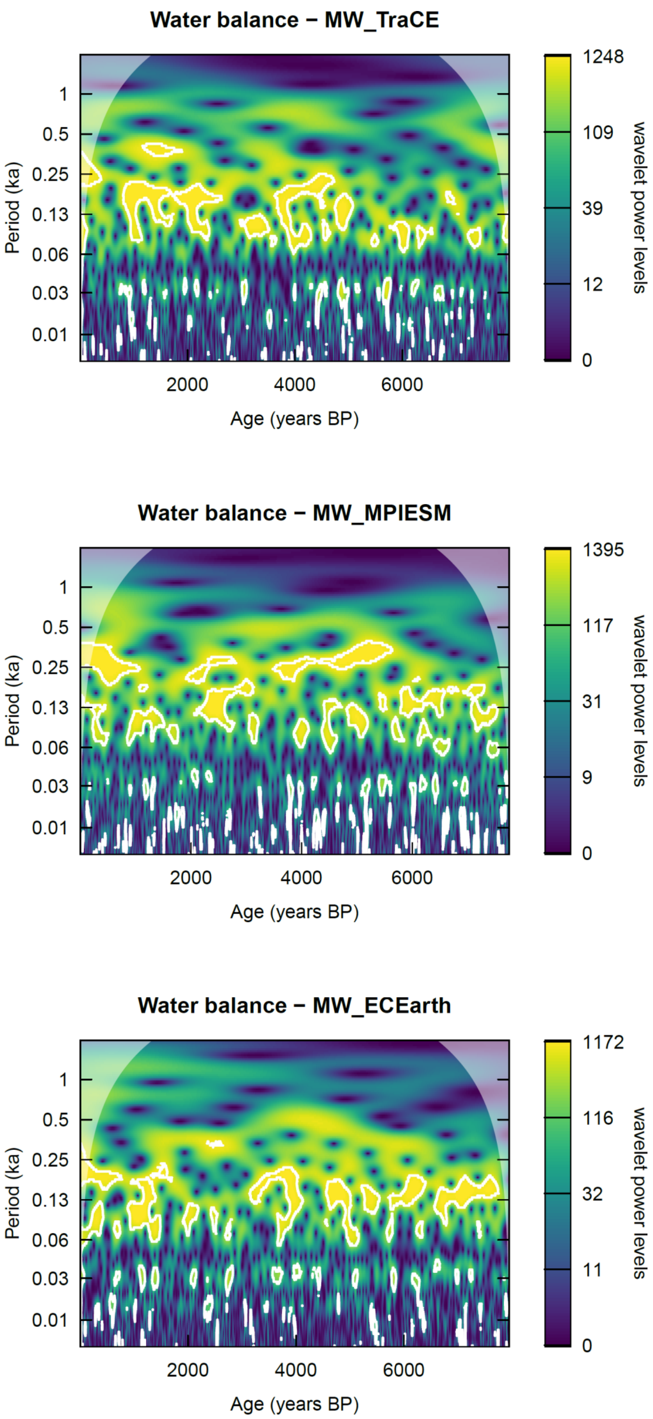

Supplementary Figure 20. As Supplementary Figure 18, but for annual water balance anomalies in the transient climate model simulations in the spatial domain of the Midwest fossil site cluster.

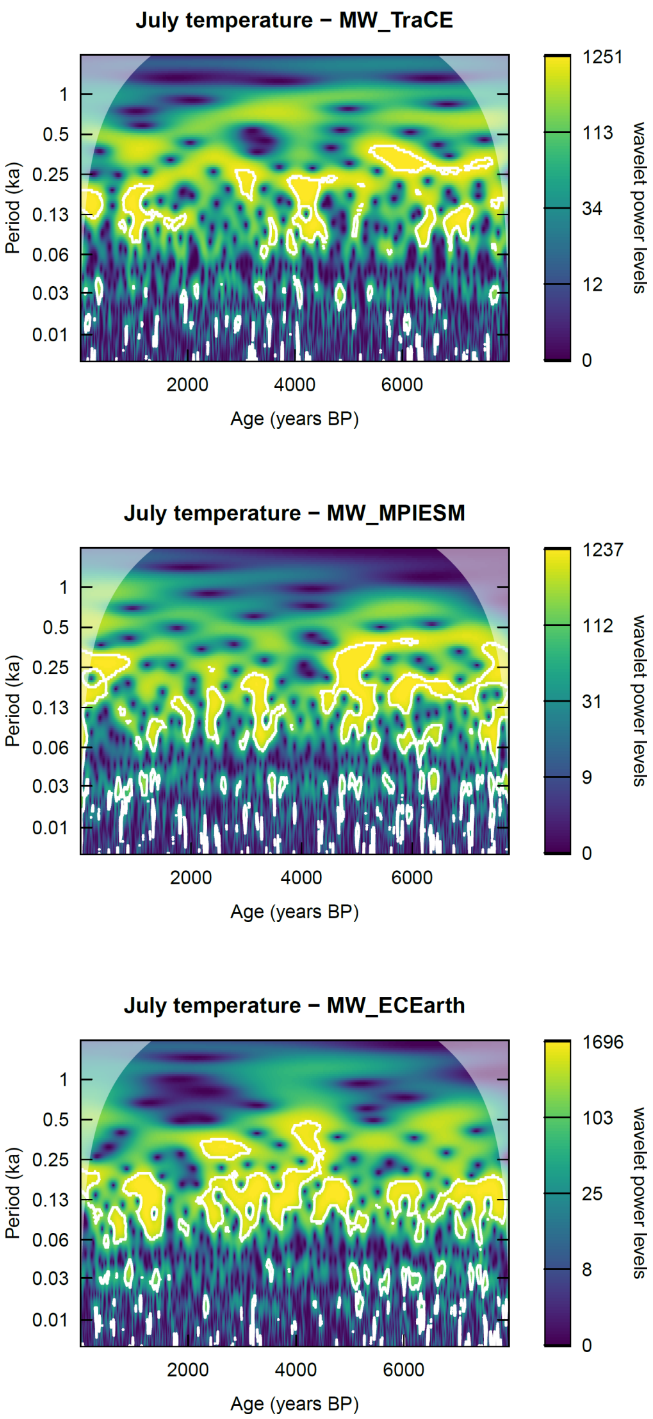

Supplementary Figure 21. As Supplementary Figure 18, but for July temperature anomalies in the transient climate model simulations in the spatial domain of the Midwest fossil site cluster.

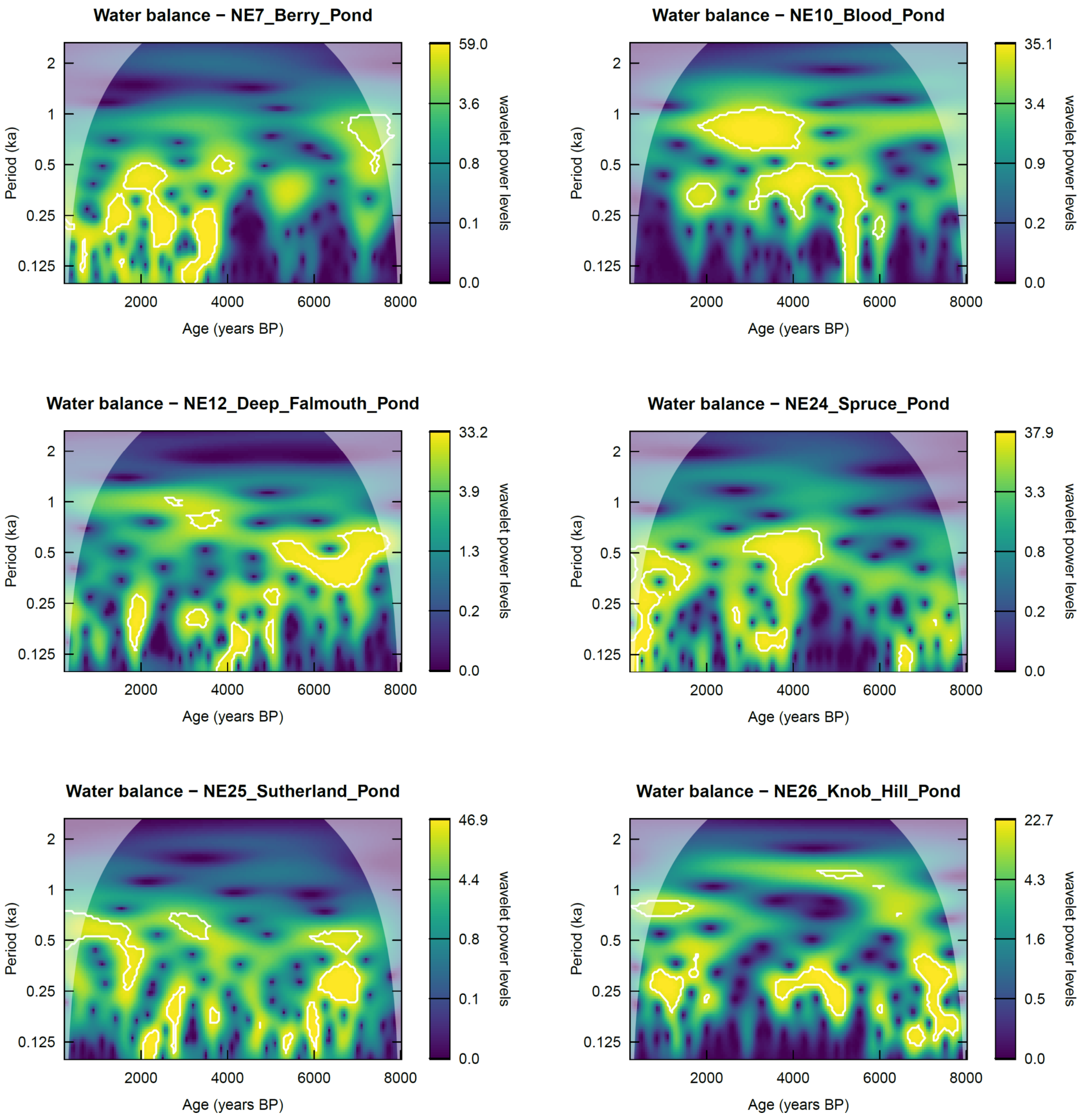

Supplementary Figure 22. As Supplementary Figure 18, but for the annual water balance reconstructions from the high-resolution fossil pollen sequences of the Northeast site cluster.

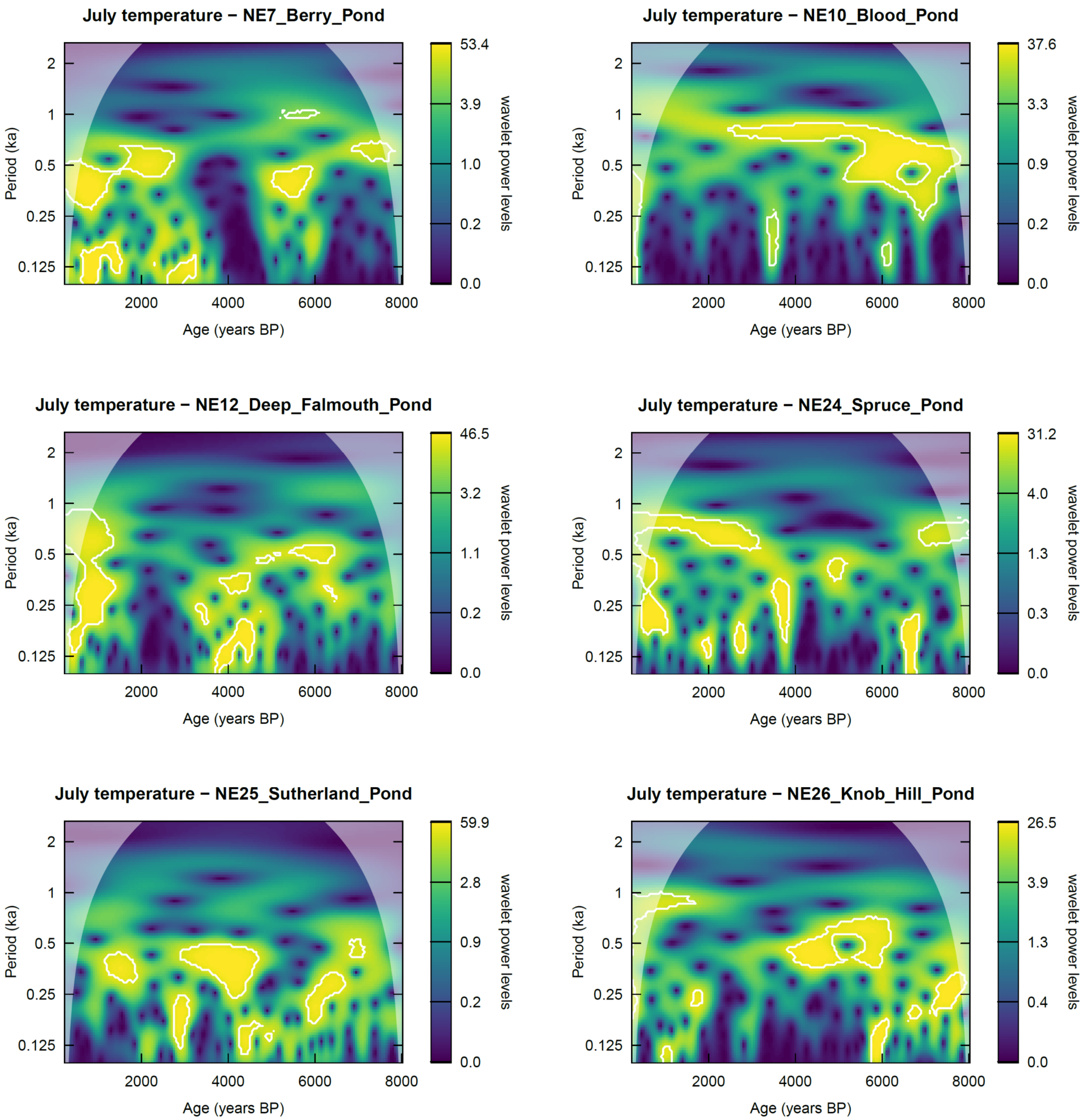

Supplementary Figure 23. As Supplementary Figure 18, but for the July temperature reconstructions from the high-resolution fossil pollen sequences of the Northeast site cluster.

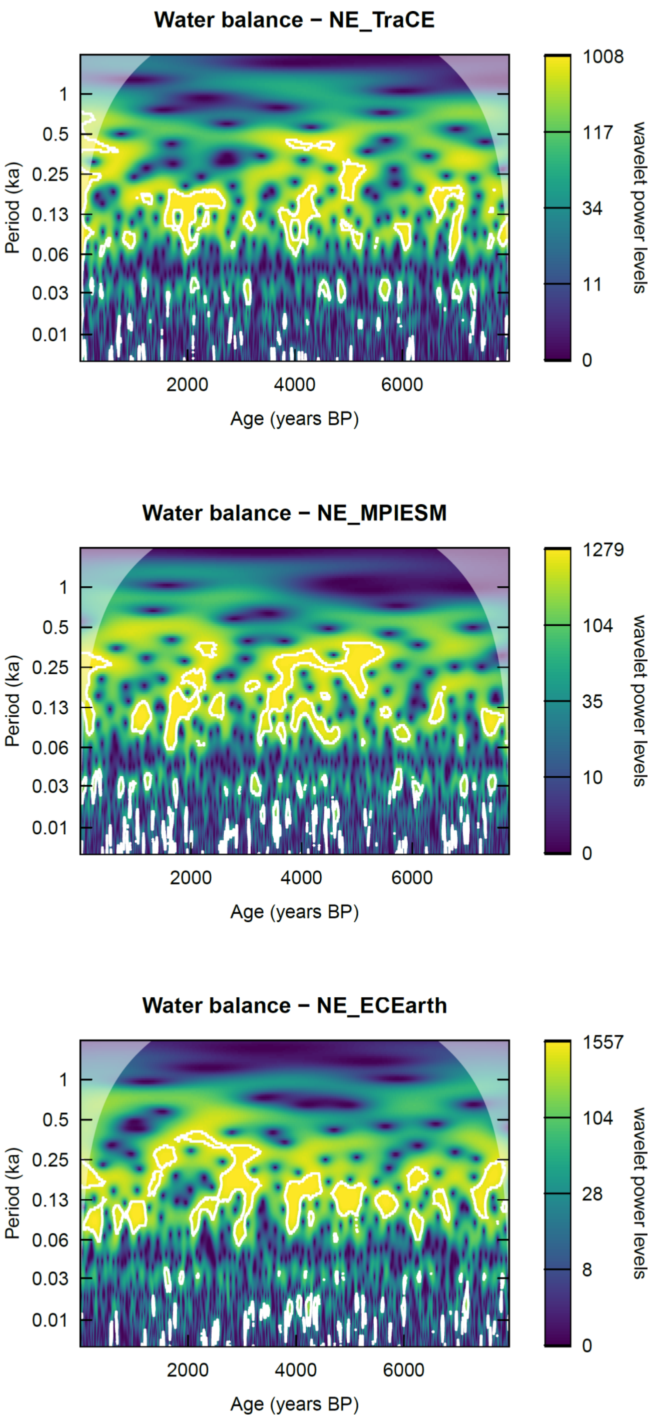

Supplementary Figure 24. As Supplementary Figure 18, but for the annual water balance anomalies in the transient climate model simulations in the spatial domain of the Northeast fossil site cluster.

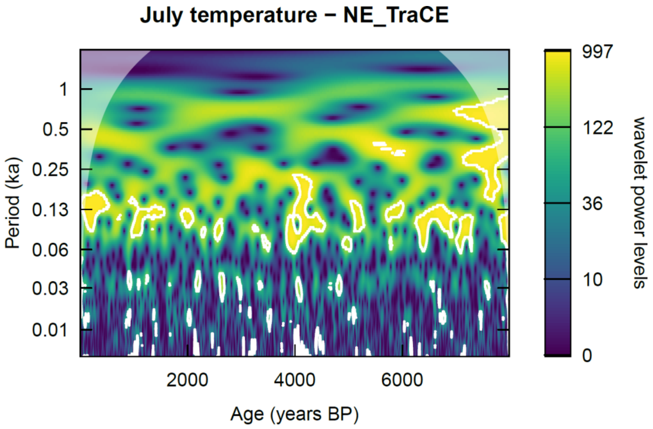

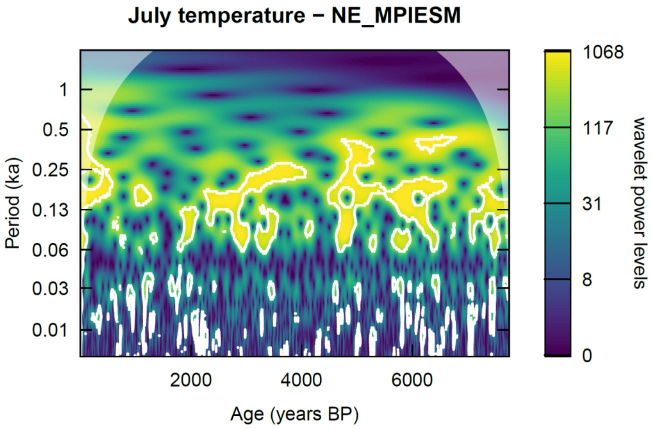

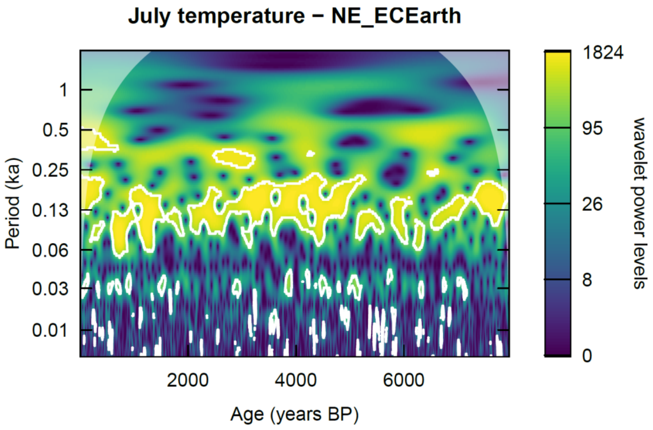

Supplementary Figure 25. As Supplementary Figure 18, but for the July temperature anomalies in the transient climate model simulations in the spatial domain of the Northeast fossil site cluster.

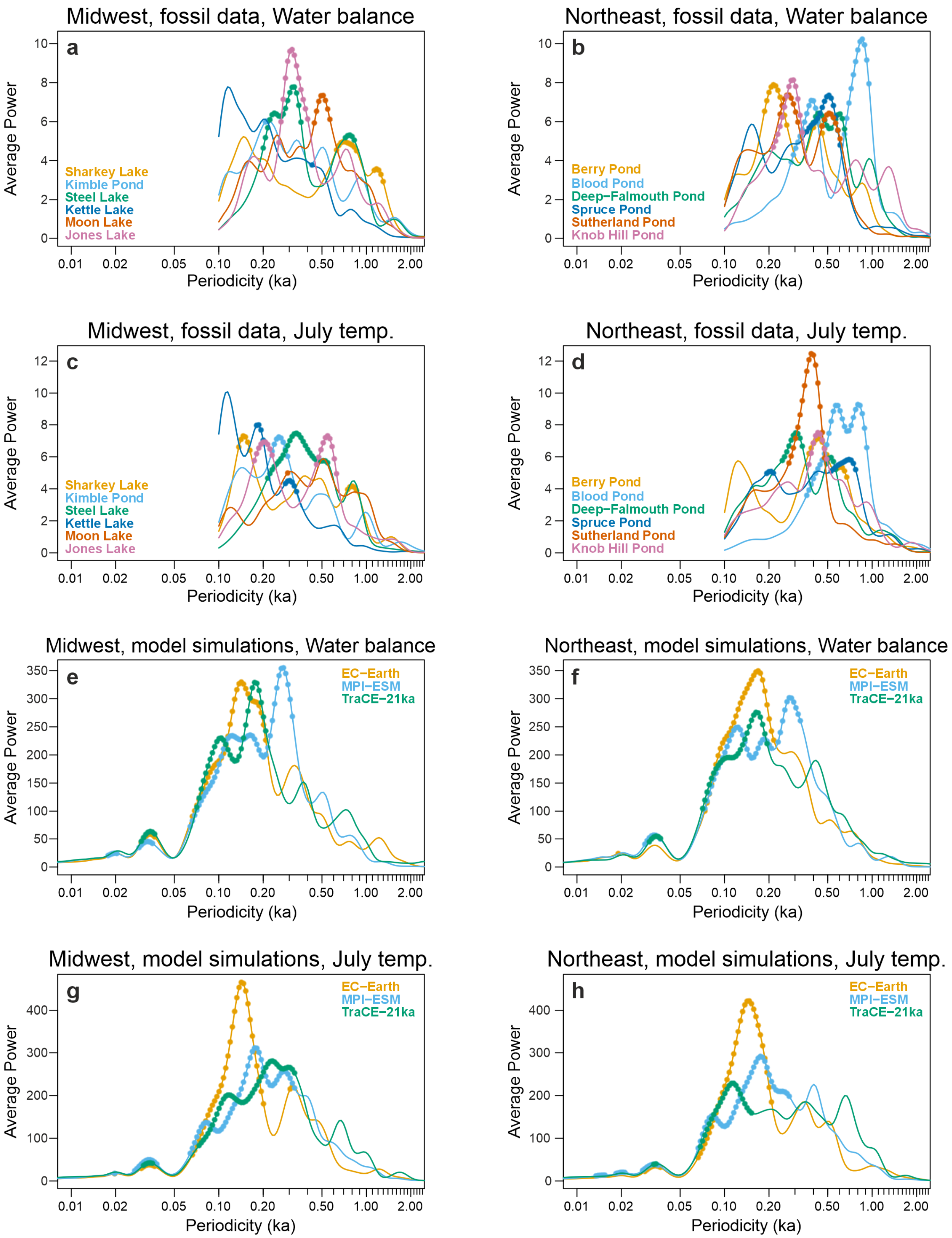

Supplementary Figure 26. Average power plots based on the wavelet analyses. The panels indicate the average power in the individual wavelet analyses (Supplementary Figures 18–25) of annual water balance and mean July temperature in reconstructions from high-resolution fossil pollen sequences (panels **a–d**) and in transient climate model simulations (**e–h**) for the Midwest and Northeast regions. The circles indicate significant power to a 95% confidence interval, considering red noise (AR-1) to describe the background variability.

Supplementary References

1 Williams, J. W. *et al.* The Neotoma Paleoecology Database, a multiproxy, international, community-curated data resource. *Quaternary Research* **89**, 156-177 (2018). https://doi.org/10.1017/qua.2017.105

2 New, M., Lister, D., Hulme, M. & Makin, I. A high-resolution data set of surface climate over global land areas. *Climate research* **21**, 1-25 (2002).

3 Van Zant, K. Late glacial and postglacial pollen and plant macrofossils from Lake West Okoboji, Northwestern Iowa. *Quaternary Research* **12**, 358-380 (1979). https://doi.org/10.1016/0033-5894(79)90034-6

4 Baker, R. G., Maher, L. J., Chumbley, C. A. & Van Zant, K. L. Patterns of Holocene environmental change in the midwestern United States. *Quaternary Research* **37**, 379-389 (1992). https://doi.org/10.1016/0033-5894(92)90074-S

5 Janssen, C. R. Myrtle Lake: a late- and post-glacial pollen diagram from northern Minnesota. *Canadian Journal of Botany* **46**, 1397-1408 (1968). https://doi.org/10.1139/b68-190

6 Janssen, C. R. Modern Pollen Assemblages and Vegetation in the Myrtle Lake Peatland, Minnesota. *Ecological Monographs* **54**, 213-252 (1984). https://doi.org/10.2307/1942662

7 Stuiver, M. Yale Natural Radiocarbon Measurements IX. *Radiocarbon* **11**, 545-658 (1969). https://doi.org/10.1017/S0033822200011413

8 Alwin, B. C. *Vegetation history of the Sugar Hills area, Itasca Co., Minnesota.*, University of Minnesota, (1982).

9 Almendinger, J. E. *Lake and groundwater paleohydrology: a groundwater model to explain past lake levels in west-central Minnesota.*, University of Minnesota, (1988).

10 Almquist-Jacobson, H., Almendinger, J. E. & Hobbie, S. Influence of terrestrial vegetation on sediment-forming processes in kettle lakes of west-central Minnesota. *Quaternary Research* **38**, 103-116 (1992). https://doi.org/10.1016/0033-5894(92)90033-F

11 Jacobson, G. L. J. *A palynological study of the history and ecology of white pine in Minnesota* Ph.D. thesis, University of Minnesota, (1975).

12 Bender, M. M., Bryson, R. A. & Baerreis, D. A. University of Wisconsin Radiocarbon Dates XIV. *Radiocarbon* **19**, 127-137 (1977). https://doi.org/10.1017/S0033822200003404

13 Jacobson, G. L. The Palaeoecology of White Pine (Pinus Strobus) in Minnesota. *Journal of Ecology* **67**, 697-726 (1979). https://doi.org/10.2307/2259121

14 Jacobson, G. L. & Grimm, E. C. A Numerical Analysis of Holocene Forest and Prairie Vegetation in Central Minnesota. *Ecology* **67**, 958-966 (1986). https://doi.org/10.2307/1939818

15 Steventon, R. L. & Kutzbach, J. E. University of Wisconsin Radiocarbon Dates XX. *Radiocarbon* **25**, 152-168 (1983). https://doi.org/10.1017/S0033822200005348

16 Hu, F. S., Wright, H. E., Jr, Ito, E. & Lease, K. Climatic effects of glacial Lake Agassiz in the midwestern United States during the last deglaciation. *Geology* **25**, 207-210 (1997). https://doi.org/10.1130/0091-7613(1997)025<0207:Ceogla>2.3.Co;2

17 Hu, F. S. *et al.* Abrupt changes in North American climate during early Holocene times. *Nature* **400**, 437-440 (1999). https://doi.org/10.1038/22728

18 Grimm, E. C. *An ecological and paleoecological study of the vegetation in the Big Woods region of Minnesota.* Ph.D. thesis, University of Minnesota, (1981).

19 Grimm, E. C. Chronology and dynamics of vegetation change in the prairie-woodland region of southern Minnesota, U.S.A. *New Phytologist* **93**, 311-350 (1983). https://doi.org/10.1111/j.1469-8137.1983.tb03434.x

20 Brugam, R. B., Grimm, E. C. & Eyster-Smith, N. M. Holocene environmental changes in Lily Lake, minnesota inferred from fossil diatom and pollen assemblages. *Quaternary Research* **30**, 53-66 (1988). https://doi.org/10.1016/0033-5894(88)90087-7

21 Stuiver, M. Climate versus changes in 13C content of the organic component of lake sediments during the Late Quarternary. *Quaternary Research* **5**, 251-262 (1975). https://doi.org/10.1016/0033-5894(75)90027-7

22 Waddington, J. C. B., Schumm, S. A. & Bradley, W. C. in *United States Contributions to Quaternary Research; Papers Prepared on the Occasion of the VIII Congress of the International Association for Quaternary Research Paris, France, 1969* Vol. 123 0 (Geological Society of America, 1969).

23 Camill, P. *et al.* Late-Glacial and Holocene Climatic Effects on Fire and Vegetation Dynamics at the Prairie-Forest Ecotone in South-Central Minnesota. *Journal of Ecology* **91**, 822-836 (2003).

24 Geiss, C. E., Umbanhowar, C. E., Camill, P. & Banerjee, S. K. Sediment magnetic properties reveal holocene climate change along the Minnesota prairie-forest ecotone. *Journal of Paleolimnology* **30**, 151-166 (2003). https://doi.org/10.1023/A:1025574100319

25 Nelson, D. M., Hu, F. S., Tian, J., Stefanova, I. & Brown, T. A. Response of C3 and C4 plants to middle-Holocene climatic variation near the prairie–forest ecotone of Minnesota. *Proceedings of the National Academy of Sciences* **101**, 562-567 (2004). https://doi.org/10.1073/pnas.0307450100

26 Tlan, J., Brown, T. A. & Hul, F. S. Comparison of varve and 14C chronologies from Steel Lake, Minnesota, USA. *The Holocene* **15**, 510-517 (2005). https://doi.org/10.1191/0959683605hl828rp
27 Wright, H. E., Stefanova, I., Tian, J., Brown, T. A. & Hu, F. S. A chronological framework for the Holocene vegetational history of central Minnesota: the Steel Lake pollen record. *Quaternary Science Reviews* **23**, 611-626 (2004). https://doi.org/10.1016/j.quascirev.2003.09.003
28 Clark, J. S. *et al.* Drought Cycles and Landscape Responses to past Aridity on Prairies of the Northern Great Plains, USA. *Ecology* **83**, 595-601 (2002). https://doi.org/10.2307/3071864
29 Brown, K. J. *et al.* Fire cycles in North American interior grasslands and their relation to prairie drought. *Proceedings of the National Academy of Sciences* **102**, 8865-8870 (2005). https://doi.org/doi:10.1073/pnas.0503621102
30 Grimm, E. C., Donovan, J. J. & Brown, K. J. A high-resolution record of climate variability and landscape response from Kettle Lake, northern Great Plains, North America. *Quaternary Science Reviews* **30**, 2626-2650 (2011). https://doi.org/10.1016/j.quascirev.2011.05.015
31 Laird, K. R., Fritz, S. C., Grimm, E. C. & Mueller, P. G. Century scale paleoclimatic reconstruction from Moon Lake, a closed-basin lake in the northern Great Plains. *Limnology and Oceanography* **41**, 890-902 (1996). https://doi.org/10.4319/lo.1996.41.5.0890
32 Jacobson, H. A. & Engstrom, D. R. Resolving the chronology of recent lake sediments: an example from Devils Lake, North Dakota. *Journal of Paleolimnology* **2**, 81-97 (1989). https://doi.org/10.1007/BF00177042
33 Teed, R., Umbanhower, C. & Camill, P. Multiproxy lake sediment records at the northern and southern boundaries of the Aspen Parkland region of Manitoba, Canada. *The Holocene* **19**, 937-948 (2009). https://doi.org/10.1177/0959683609336569
34 Ritchie, J. C. Contributions to the Holocene Paleoecology of Westcentral Canada: I. The Riding Mountain Area. *Canadian Journal of Botany* **42**, 181-196 (1964). https://doi.org/10.1139/b64-018
35 Ritchie, J. C. Absolute pollen frequencies and carbon-14 age of a section of Holocene Lake sediment from the Riding Mountain area of Manitoba. *Canadian Journal of Botany* **47**, 1345-1349 (1969). https://doi.org/10.1139/b69-192
36 Ritchie, J. C. The late-Quaternary vegetational history of the Western Interior of Canada. *Canadian Journal of Botany* **54**, 1793-1818 (1976). https://doi.org/10.1139/b76-194
37 Moos, M. T. *Tracking long-term Holocene climate trends in Lake 239 (experimental lakes area, NW Ontario) using diatoms, pollen, and charcoal* Ph.D. thesis, Queen's University, (2010).
38 Nelson, D. M., Hu, F. S., Grimm, E. C., Curry, B. B. & Slate, J. E. The Influence of Aridity and Fire on Holocene Prairie Communities in the Eastern Prairie Peninsula. *Ecology* **87**, 2523-2536 (2006).
39 Saunders, J. J. *et al.* Paradigms and proboscideans in the southern Great Lakes region, USA. *Quaternary International* **217**, 175-187 (2010). https://doi.org/10.1016/j.quaint.2009.07.031
40 Schubert, B. W., Graham, R. W., McDonald, H. G., Grimm, E. C. & Stafford, T. W. Latest Pleistocene paleoecology of Jefferson's ground sloth (Megalonyx jeffersonii) and elk-moose (Cervalces scotti) in northern Illinois. *Quaternary Research* **61**, 231-240 (2004). https://doi.org/10.1016/j.yqres.2003.10.005
41 Curry, B. B. *et al.* Quaternary geology, geomorphology, and climatic history of Kane County, Illinois., (Illinois State Geological Survey, Champaign, Illinois, USA, 1999).
42 Curry, B. B., Grimm, E. C., Slate, J. E., Hansen, B. C. S. & Konen, M. E. The late-glacial and early Holocene geology, paleoecology, and paleohydrology of the Brewster Creek site, a proposed wetland restoration site, Pratt's Wayne Woods Forest Preserve, and James “Pate” Philip State Park, Bartlett, Illinois. . (Illinois State Geological Survey, Champaign, Illinois, USA, 2007).
43 Wang, Y. *et al.* Pronounced variations in Fagus grandifolia abundances in the Great Lakes region during the Holocene. *The Holocene* **26**, 578-591 (2016). https://doi.org/10.1177/0959683615612586
44 Brubaker, L. B. Postglacial forest patterns associated with till and outwash in northcentral Upper Michigan. *Quaternary Research* **5**, 499-527 (1975). https://doi.org/10.1016/0033-5894(75)90013-7
45 Davis, M. B., Schwartz, M. W. & Woods, K. Detecting a Species Limit from Pollen in Sediments. *Journal of Biogeography* **18**, 653-668 (1991). https://doi.org/10.2307/2845547
46 Rasmussen, J. B. *Pollen stratigraphy and vegetational history of Cub Lake, Kalkaska County, Michigan*, Central Michigan University, (1982).
47 Brugam, R. B., Giorgi, M., Sesvold, C., Johnson, S. M. & Almos, R. Holocene Vegetation History in the Sylvania Wilderness Area of the Western Upper Peninsula of Michigan. *The American Midland Naturalist* **137**, 62-71 (1997). https://doi.org/10.2307/2426755
48 Brugam, R. B., Owen, B. & Kolesa, L. Continental-scale climate forcing factors and environmental change at Glimmerglass Lake in the upper Peninsula of Michigan. *The Holocene* **14**, 807-817 (2004). https://doi.org/10.1191/0959683604hl761rp
49 Ewing, H. A. *Ecosystem development and response to climatic change: a comparative study of forest-lake ecosystems on different substrates* Ph.D. thesis, University of Minnesota, (2000).

50 Ewing, H. A. The Influence of Substrate on Vegetation History and Ecosystem Development. *Ecology* **83**, 2766-2781 (2002). https://doi.org/10.2307/3072014
51 Booth, R. K., Jackson, S. T. & Thompson, T. A. Paleoecology of a Northern Michigan Lake and the Relationship among Climate, Vegetation, and Great Lakes Water Levels. *Quaternary Research* **57**, 120-130 (2002). https://doi.org/10.1006/qres.2001.2288
52 Woods, K. D. & Davis, M. B. Paleoecology of Range Limits: Beech in the Upper Peninsula of Michigan. *Ecology* **70**, 681-696 (1989). https://doi.org/10.2307/1940219
53 Manny, B. A., Wetzel, R. G. & Bailey, R. E. Paleolimnological sedimentation of organic carbon, nitrogen, phosphorous, fossil pigments, pollen, and diatoms in a hypereutrophic, hardwater lake: a case history of eutrophication. *Polskie Archiwum Hydrobiologii* **25**, 243-267 (1978).
54 Maher Jr, L. J. in *Quaternary history of the Driftless Area* Vol. 5 (eds J. C. Knox, L. Clayton, & D. M. Mickelson) 119-135 (University of Wisconsin-Extension, Geological and Natural History Survey, 1982).
55 Spigel, K. M. *Erosion and sedimentation history of Emrick Lake, south-central Wisconsin, in response to Holocene environmental change* Ph.D. thesis, University of Wisconsin, (2006).
56 Urban, M. A. *Forest responses to late-Holocene climate change in north-central Wisconsin: a comparative plant macrofossil study of two adjacent lake* M.Sc. thesis, University of Wyoming, (2008).
57 Webb, S. L. *The Holocene extension of the range of American Beech (Fagus grandifolia) into Wisconsin: paleoecological evidence for long-distance seed dispersal*, University of Minnesota, (1983).
58 Gaudreau, D. C. *Late-Quaternary vegetational history of the northeast: paleoecological implications of topographic patterns in pollen distributions.* Ph.D. thesis, Yale University, (1986).
59 Steventon, R. L. & Kutzbach, J. E. University of Wisconsin Radiocarbon Dates XXI. *Radiocarbon* **26**, 135-147 (1984). https://doi.org/10.1017/S0033822200006494
60 Steventon, R. L. & Kutzbach, J. E. University of Wisconsin Radiocarbon Dates XXIII. *Radiocarbon* **28**, 1206-1223 (1986). https://doi.org/10.1017/S003382220002021X
61 Oswald, W. W. *et al.* Subregional variability in the response of New England vegetation to postglacial climate change. *Journal of Biogeography* **45**, 2375-2388 (2018). https://doi.org/10.1111/jbi.13407
62 Almquist-Jacobson, H. & Sanger, D. Holocene climate and vegetation in the Milford drainage basin, Maine, U.S.A., and their implications for human history. *Vegetation History and Archaeobotany* **4**, 211-222 (1995). https://doi.org/10.1007/BF00235752
63 Nurse, A. M. *12,000-year record of lake-level and vegetative change at Mathews Pond, Piscataquis County, Maine, USA* Ph.D. thesis, University of Maine, (2003).
64 Huvane, J. K. & Whitehead, D. R. The paleolimnology of North Pond: watershed-lake interactions. *Journal of Paleolimnology* **16**, 323-354 (1996). https://doi.org/10.1007/BF00207576
65 Whitehead, D. & Crisman, T. L. Paleolimnological studies of small New England (USA) ponds. Part I. Late-glacial and postglacial trophic oscillations. *Polskie Archiwum Hydrobiologii* **25**, 75 (1978).
66 Suter, S. Late-glacial and Holocene vegetation history in southeastern Massachusetts: a 14,000 year pollen record. *Current Research in the Pleistocene* **2**, 87-89 (1985).
67 Toney, J. L., Rodbell, D. T. & Miller, N. G. Sedimentologic and palynologic records of the last deglaciation and Holocene from Ballston Lake, New York. *Quaternary Research* **60**, 189-199 (2003). https://doi.org/10.1016/S0033-5894(03)00093-0
68 Ibe, R. A. *Quaternary palynology of five lacustrine deposits in the Catskill Mountain region of New York*, New York University, (1982).
69 Maenza-Gmelch, T. E. Holocene vegetation, climate, and fire history of the Hudson Highlands, southeastern New York, USA. *The Holocene* **7**, 25-37 (1997). https://doi.org/10.1177/095968369700700103
70 Maenza-Gmelch, T. E. Vegetation, climate, and fire during the late-glacial–Holocene transition at Spruce Pond, Hudson Highlands, southeastern New York, USA. *Journal of Quaternary Science* **12**, 15-24 (1997). https://doi.org/10.1002/(SICI)1099-1417(199701/02)12:1<15::AID-JQS283>3.0.CO;2-T
71 Maenza-Gmelch, T. E. Late-glacial – early Holocene vegetation, climate, and fire at Sutherland Pond, Hudson Highlands, southeastern New York, U.S.A. *Canadian Journal of Botany* **75**, 431-439 (1997). https://doi.org/10.1139/b97-045
72 Mott, R. J. Late-Pleistocene and Holocene palynology in southeastern Québec. *Géographie physique et Quaternaire* **31**, 139-149 (1977). https://doi.org/10.7202/1000060ar
73 Williams, J. W. & Shuman, B. Obtaining accurate and precise environmental reconstructions from the modern analog technique and North American surface pollen dataset. *Quaternary Science Reviews* **27**, 669-687 (2008). https://doi.org/10.1016/j.quascirev.2008.01.004
74 Elith, J., Leathwick, J. R. & Hastie, T. A working guide to boosted regression trees. *Journal of Animal Ecology* **77**, 802-813 (2008). https://doi.org/10.1111/j.1365-2656.2008.01390.x
75 Telford, R. J. & Birks, H. J. B. Evaluation of transfer functions in spatially structured environments. *Quaternary Science Reviews* **28**, 1309-1316 (2009). https://doi.org/10.1016/j.quascirev.2008.12.020
